# From transient transcriptome responses to disturbed neurodevelopment: role of histone acetylation and methylation as epigenetic switch between reversible and irreversible drug effects

**DOI:** 10.1007/s00204-014-1279-6

**Published:** 2014-06-17

**Authors:** Nina V. Balmer, Stefanie Klima, Eugen Rempel, Violeta N. Ivanova, Raivo Kolde, Matthias K. Weng, Kesavan Meganathan, Margit Henry, Agapios Sachinidis, Michael R. Berthold, Jan G. Hengstler, Jörg Rahnenführer, Tanja Waldmann, Marcel Leist

**Affiliations:** 1 Doerenkamp-Zbinden Chair for In Vitro Toxicology and Biomedicine, University of Konstanz, Box 657, 78457 Constance, Germany; 2 Department of Statistics, TU Dortmund, Dortmund, Germany; 3 Chair for Bioinformatics and Information Mining, University of Konstanz, Constance, Germany; 4 Konstanz Research School Chemical Biology, University of Konstanz, Constance, Germany; 5 OÜ Quretec, 51003 Tartu, Estonia; 6 Institute of Neurophysiology, University of Cologne, 50931 Cologne, Germany; 7 Leibniz Research Centre for Working Environment and Human Factors (IfADo), 44139 Dortmund, Germany

**Keywords:** Valproic acid, Embryonic stem cell, Histone deacetylase, Developmental toxicity, Histone modification

## Abstract

The superordinate principles governing the transcriptome response of differentiating cells exposed to drugs are still unclear. Often, it is assumed that toxicogenomics data reflect the immediate mode of action (MoA) of drugs. Alternatively, transcriptome changes could describe altered differentiation states as indirect consequence of drug exposure. We used here the developmental toxicants valproate and trichostatin A to address this question. Neurally differentiating human embryonic stem cells were treated for 6 days. Histone acetylation (primary MoA) increased quickly and returned to baseline after 48 h. Histone H3 lysine methylation at the promoter of the neurodevelopmental regulators PAX6 or OTX2 was increasingly altered over time. Methylation changes remained persistent and correlated with neurodevelopmental defects and with effects on PAX6 gene expression, also when the drug was washed out after 3–4 days. We hypothesized that drug exposures altering only acetylation would lead to reversible transcriptome changes (indicating MoA), and challenges that altered methylation would lead to irreversible developmental disturbances. Data from pulse-chase experiments corroborated this assumption. Short drug treatment triggered reversible transcriptome changes; longer exposure disrupted neurodevelopment. The disturbed differentiation was reflected by an altered transcriptome pattern, and the observed changes were similar when the drug was washed out during the last 48 h. We conclude that transcriptome data after prolonged chemical stress of differentiating cells mainly reflect the altered developmental stage of the model system and not the drug MoA. We suggest that brief exposures, followed by immediate analysis, are more suitable for information on immediate drug responses and the toxicity MoA.

**Electronic supplementary material:**

The online version of this article (doi:10.1007/s00204-014-1279-6) contains supplementary material, which is available to authorized users.

## Introduction

Toxicogenomics data, systems biology, and the use of human stem cell-based systems are expected to change the ways by which toxicological information will be obtained and interpreted in the future (Hartung et al. 2012; Robinson et al. 2012a; Robinson and Piersma 2013; Waters and Fostel 2004; Wobus and Loser 2011). This is in line with a ‘proposed shift from primarily in vivo animal experimentation to in vitro assays and computational modeling for toxicity assessment,’ as suggested by the lead scientists of US national research agencies (Collins et al. 2008). It also follows the ‘vision for a new toxicology of the twenty-first century’ as promoted by the National Research Council (Andersen and Krewski 2010; NRC 2007). A key assumption for this vision is that it will be possible to define pathways of toxicity, i.e., a drug mode of action (MoA), linking molecular initiating events to a final adverse outcome. This requires an establishment of a ‘systems toxicology’ that models the pathophysiology of the body with computational tools to understand mechanisms of toxicity, similar to systems biology (Hartung et al. 2012).

In the field of developmental toxicology, transcriptome data have been used to infer information on the MoA of chemicals (Colleoni et al. 2011; Hermsen et al. 2013; Vojnits et al. 2012). It is expected that such approaches will lead to major conceptual advances, especially for the use of the emerging technology of differentiating stem cell systems (Crofton et al. 2012; Theunissen et al. 2012; Zimmer et al. 2011). However, more fundamental work is required to understand how experiments need to be designed and interpreted in this field. In contrast to mature tissues or cells, model systems of development do not have a stable baseline, i.e., the transcriptome changes over time, also without toxic stimulus. Moreover, initial exposure to a toxicant may trigger secondary effects even in the absence of the stimulus (Balmer et al. 2012). To avoid indirect effects in toxicogenomics measurements, sampling only few hours after compound exposure has been suggested (Jergil et al. 2011). However, the sensitivity and response to toxicants of a dynamically differentiating system can be different at different times (van Dartel et al. 2009). For instance, in a study of retinoic acid teratogenicity, compound-induced transcriptome changes differed between sampling time points (Robinson et al. 2012b). Such timing effects of toxicants may be due to interference with specific waves of gene regulation. For instance, neurally differentiating mESC showed several of such waves of gene expression, which determined windows of sensitivity to toxicants (Abranches et al. 2009; Zimmer et al. 2011).

Alteration of histone deacetylase (HDAC) activity has been associated with several long-term health consequences, ranging from Alzheimer’s disease (Graff et al. 2012), over toluene poisoning (Sanchez-Serrano et al. 2011) to general teratogenic mechanisms (Menegola et al. 2012). Specific HDAC inhibitor (HDACi) drugs have been particularly well characterized. For instance, the broad-spectrum HDACi valproic acid (VPA), normally used to treat epilepsies, causes the fetal valproate syndrome and has been suspected to trigger autism (Dufour-Rainfray et al. 2010; Jentink et al. 2010; Meador et al. 2009). It triggers gene activation within few hours in several tumor or stem cell lines (Jergil et al. 2009, 2011), and it is well recognized that epigenetic modifications are related to the developmental neurotoxicity of the drug. Most studies addressing the latter mechanism have concentrated on transcription-activating histone modifications such as acetylation of histone 3 at lysine 9 (H3K9Ac) or methylation of histone 3 at lysine 4 (H3K4me) (Hezroni et al. 2011; Marinova et al. 2011; Tung and Winn 2010), and the changes have been found to be reversible after drug withdrawal (Boudadi et al. 2013).

In previous work, we established a model of early neural differentiation of hESC that allowed the identification of developmental toxicants (Balmer et al. 2012; Krug et al. 2013). We found that prolonged exposure to the two HDACi trichostatin A (TSA) or VPA altered the expression of several marker genes in a similar way. However, we also observed that various hESC-based test systems differed strongly in their transcriptome response to VPA (Krug et al. 2013). This latter finding triggered the key question of this study: Are the observations on altered transcriptome patterns in developmental toxicity studies indeed a reflection of a compounds primary MoA? As alternative hypothesis, we examined whether the data rather reflect an altered cellular phenotype that would result from disturbed differentiation and that would become independent of the continued presence of drug after some time. This was addressed by transcriptome analysis after pulsed drug exposure. A further key question was how a direct, but reversible effect of short toxicant exposure was switched to a persistent adverse effect, reflected by wrong differentiation after a longer drug exposure. This was addressed by studies of the time dependence of histone modifications. Histone methylations at the promoters of key neurodevelopmental genes were considered as potential persistence detectors responsible for switching a short-term cellular adaptation to permanent toxicity.

## Materials and methods

### Materials

Gelatine, putrescine, selenium, progesterone, apotransferin, glucose, insulin, valproic acid, and trichostatin A were obtained from Sigma (Steinheim, Germany). Accutase was from PAA (Pasching, Austria). FGF-2 (basic fibroblast growth factor), noggin, and sonic hedgehog were obtained from R&D Systems (Minneapolis, MN, USA). Y-27632, SB-43154, and dorsomorphin dihydrochloride were from Tocris Bioscience (Bristol, UK). MatrigelTM was from BD Biosciences (Massachusetts, USA). All cell culture reagents were from Gibco/Invitrogen (Darmstadt, Germany) unless otherwise specified.

### Neuroepithelial differentiation

Human embryonic stem cells (hESC) (H9 from WiCells, Madison, WI, USA) were differentiated as described in detail earlier (Chambers et al. 2009). Briefly, dual SMAD inhibition was used to prevent BMP and TGF signaling and thus to achieve a highly selective neuroectodermal lineage commitment. For handling details, see supplemental methods of Balmer et al. (2012). If not stated otherwise, treatment with trichostatin A (TSA) was done with a concentration of 10 nM and treatment with valproic acid (VPA) was done with a concentration of 600 µM.

### Quantitative real-time PCR (qPCR) and microarray analysis

For qPCR analysis, cells were lysed at indicated days of differentiation in TriFast™ (Peqlab, Germany). Total RNA was isolated according to the manufacturer’s instruction, and cDNA was produced using the iScript Kit from BioRad (iScript™ Reverse Transcription Supermix for RT-qPCR, BioRad). Quantitative real-time PCR (qPCR) was performed on a BioRad Light Cycler (Biorad, München, Germany), and transcript levels were quantified as described earlier (Balmer et al. 2012). The sequences of specific primers are given in Fig. S11.

Affymetrix chip-based DNA microarray analysis (Human Genome U133 plus 2.0 arrays) was performed as described earlier (Krug et al. 2013). The data were analyzed for differential expression using the Konstanz Information Miner open source software [KNIME; www.knime.org (Berthold et al. 2007)]. The raw data were preprocessed using robust multiarray analysis (RMA) (Smyth 2005). Background correction, quantile normalization, and summarization were applied to all expression data samples, using the RMA function from the *affy* package of Bioconductor (Gautier et al. 2004; Gentleman et al. 2004). The *limma* package (R & Bioconductor) was used to identify differentially expressed genes using indicated groups as control. The moderated *t* statistics was applied in a pairwise fashion (each treatment was compared to its own control) and was used for assessing the raw significance of differentially expressed genes. Then, final *p* values were derived using the Benjamini–Hochberg (BH) method to control the false discovery rate (FDR) (Benjamini and Hochberg 1995) due to multiple hypothesis testing. Transcripts with FDR adjusted *p* value of ≤0.05 and fold change values >1.5 or <2/3 were considered significantly regulated, if not stated otherwise in the figure legend. For Fig. S5, numbers of PS changed during development (D-genes) were calculated relative to hESC. These data were obtained from four independent replicates, and they were considered significant if the Benjamini–Yekutieli (BY)-adjusted *p* value was <0.01 and the FC was >1.5 or <2/3. For Figs. 3, 4, 5, and 6, numbers of PS changed by the treatment were calculated relative to untreated controls lysed at the same day as the treated samples (T6h to C6h, T4d to C4d and T6d, early pulse (EP), medium pulse (MP), and late pulse (LP) to C6d). Data were obtained from four independent replicates and chosen if the BH-adjusted *p* value was <0.05 and FC was >1.5 or FC <2/3.

The principal component analysis (PCA) was based on 500 PS with the highest variance.

### Western blot

Western blot was performed exactly as previously described (Balmer et al. 2012). For quantification, the signal intensity of H3Ac was normalized to total H3, and acetylated α-tubulin was normalized to total α-tubulin for every time point. These normalized values were then displayed relative to untreated controls at the respective time points. Western blots of PAX6 and OTX2 were quantified using ImageJ. Detailed information on used antibodies is given in Fig. S12.

### Chromatin immunoprecipitation (ChIP)

**Fig. 1:**
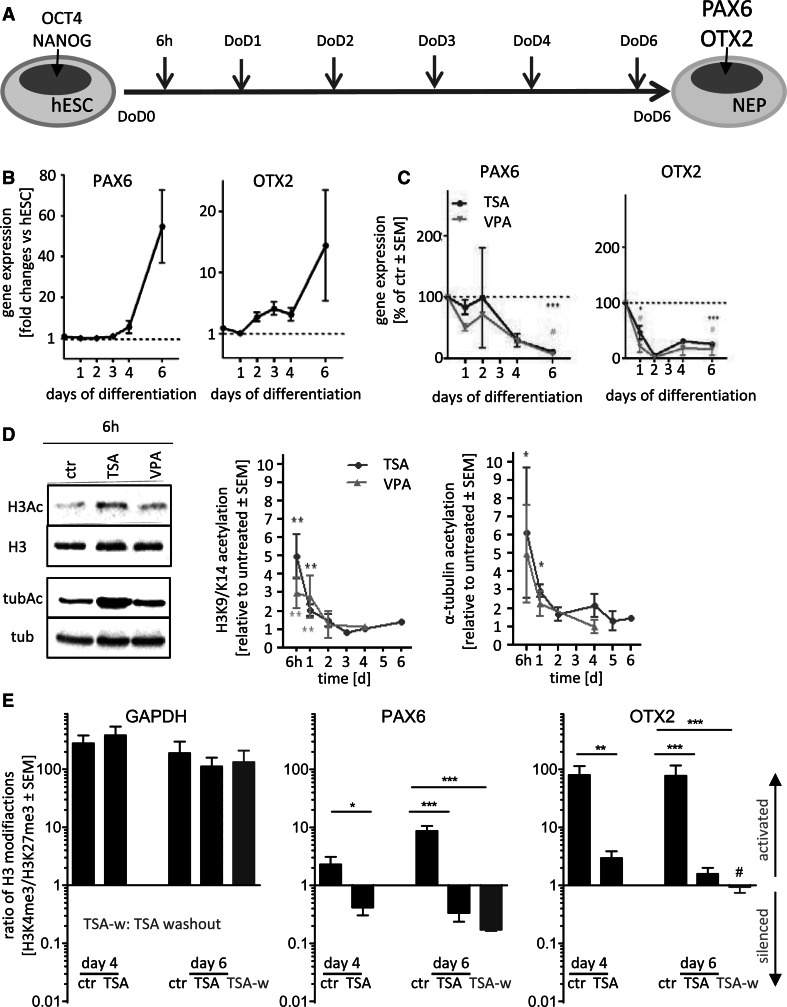
Gene expression and histone methylation patterns of the neuroectodermal marker genes PAX6 and OTX2. **a** For all experiments, human embryonic stem cells (hESC) were differentiated to neuroepithelial precursor cells (NEP). Marker genes for hESC and NEP at the time points of analysis are indicated in the differentiation scheme. **b** Samples were taken at the indicated days of differentiation (DoD), and transcript levels of marker genes of neural differentiation were determined by RT-qPCR. Data (gene expression relative to hESC) are mean ± SEM of 3–5 experiments. **c** The differentiation was performed in the presence of non-cytotoxic concentrations of TSA (10 nM) or VPA (0.6 mM). At the indicated time points, transcript levels were determined by RT-qPCR. Data (expressed relative to control differentiated for the same time in the absence of drug) are mean ± SEM of 3–5 experiments. **d** Differentiating cells were treated with TSA or VPA for different time periods, and protein acetylation was analyzed by Western blots (WB) with antibodies specific for acetylated histone H3 (H3Ac) or acetylated α-tubulin (tubAc). WB against total histone 3 (H3) or α-tubulin (tub) was performed for normalization. One representative blot for the 6-h time point is displayed. The *graphs* are based on densitometric analysis of WB from three independent experiments. The levels of acetylated protein are given relative to untreated controls differentiated for the same time period. Data are mean ± SEM of three experiments. **e** Differentiating cell was treated with TSA (10 nM) for 4 days (day 4), 6 days (day 6), and for 4 days followed by a 2-day washout of the drug (TSA-w, *purple bars*). Chromatin immunoprecipitation (ChIP) was performed with antibodies specific for H3K4me3 or H3K27me3. Samples were taken on day 4 of differentiation (day 4) or on day 6 of differentiation (day 6; TSA-w). The figure displays the ratio of the enrichment factors for H3K4me3 and H3K27me3 (individual values are found in supplemental files). A ratio >1 points to open chromatin (more H3K4me3) and a ratio <1 suggests a more silenced chromatin (more H3K27me3). *Hash symbol* indicates a bivalent state. Data are mean ± SEM of three experiments. *p < 0.05; **p < 0.01; ***p < 0.01 (color figure online)

**Fig. 2:**
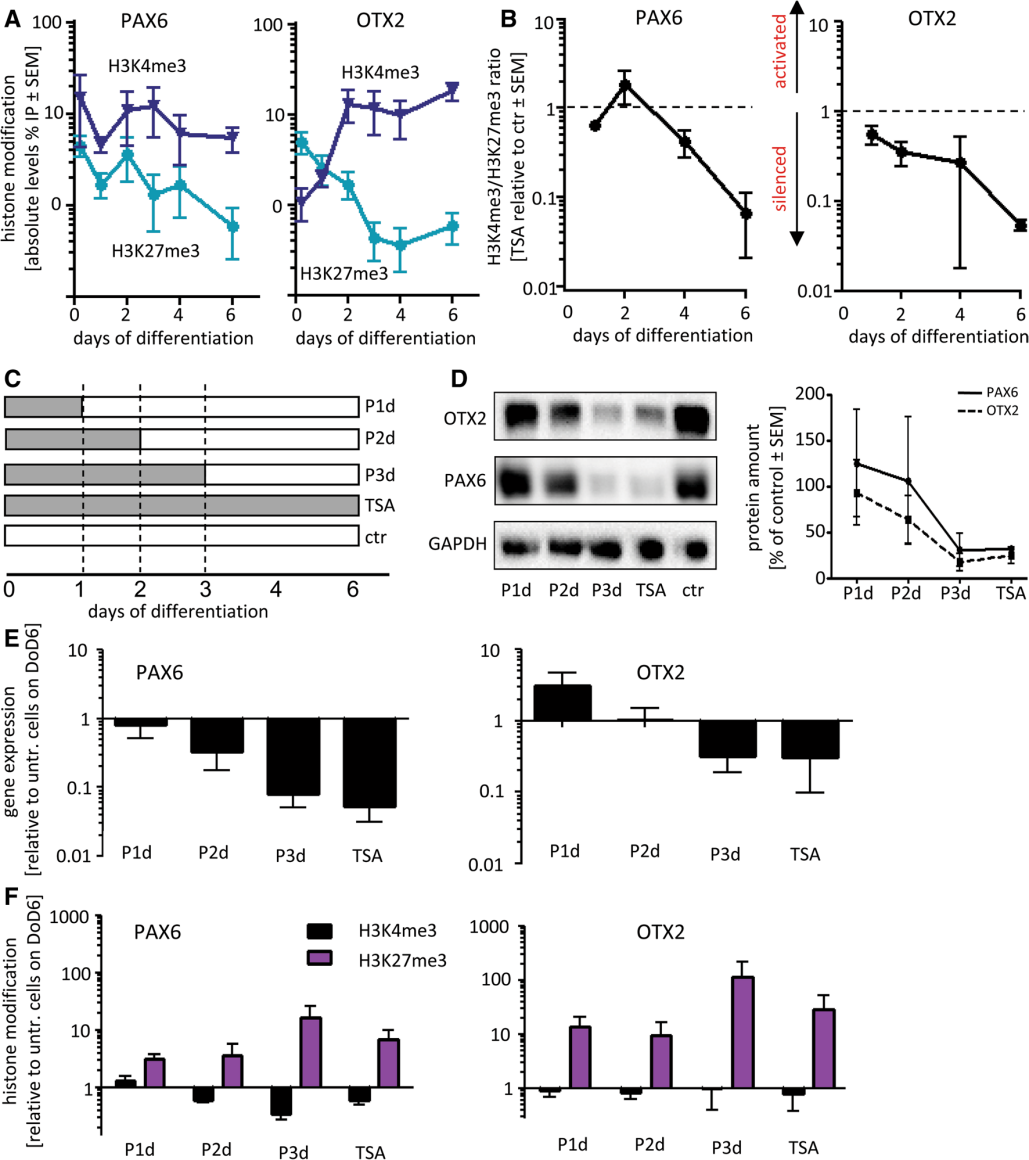
Consequences of different drug washout periods for gene expression and histone methylation patterns. For all experiments, hESC were differentiated to NEP. **a** Samples for chromatin immunoprecipitation (ChIP) were prepared at the indicated days of differentiation. ChIP was performed with antibodies specific for H3K4me3 or H3K27me3 or control IgG. The enrichment factors of OTX2 and PAX6 promoter sequences are given as % input for H3K4me3 (*dark blue*) and H3K27me3 (*light blue*). Data are mean ± SEM of three independent cell preparations. **b** Differentiating cells were treated with TSA (10 nM) for the indicated time periods, and ChIP was performed with the same antibodies as described in **a**. The ratio of enrichment factors of H3K4me3 and H3K27me3 was calculated as measure of chromatin opening. Data are given relative to values of untreated control cells at the same time point (*n* = 3). **c** Scheme of experimental treatment and sampling for the following experiments. *Gray bars* indicate the period of drug exposure (e.g., P2d: pulsed drug treatment for 2 days) with 10 nM TSA, and *white bars* indicate medium without TSA. All samples were analyzed on day 6 of differentiation for each treatment scenario. **d** Protein levels of PAX6 or OTX2 were determined by Western blot, and relative (vs ctr.) protein levels (*n* = 3) were quantified. **e** Transcript levels of PAX6 and OTX2 were determined. They are expressed relative to untreated control on DoD6 (ctr). **f** ChIP was performed for H3K27me3 (*purple*) or H3K4me3 (*black*) on promoter regions of PAX6 and OTX2, and enrichment factors were calculated relative to ChIP with control IgG. Then, these data were normalized to the values obtained for control cells (ctr). For instance, on day 6 of the differentiation, H3K27me3 was 15-fold higher in cells treated for 1 day with TSA and then left in control medium (P1d), compared to cells that were differentiated under control conditions. Data of **d**–**f** are mean ± SEM of 3–5 experiments (color figure online)

Chromatin immunoprecipitation (ChIP) assays on native chromatin (N-ChIP) (Fig. 1) were performed according to established protocols (Umlauf et al. 2004). Details and adaptations were exactly as described previously in detail (Balmer et al. 2012). ChIP assays on cross-linked chromatin (X-ChIP) (Fig. 2) were performed according to Kamieniarz and colleagues and adapted to our differentiating cells (Kamieniarz et al. 2012). Briefly, cells were trypsinized and resuspended in 1 % formaldehyde in medium. The cross-link was stopped after 10 min by 125 mM Tris, pH 7.5. Cellular suspensions were centrifuged, washed once in PBS and once in L1 buffer (2 mM EDTA, 0.1 % NP-40, 10 % glycerol, 25 mM Tris, pH 8), and finally resuspended in L2 buffer (10 mM EDTA pH 8, 1 % SDS, 50 mM Tris, pH 8) to a final concentration of 2 × 10^6^ cells/ml. Chromatin was sonicated on a Bioruptor ^®^ sonifier device (Diagenode, Belgium) by 30 steps of 30/30 s ON/OFF cycles to get a fragment size between 300 and 700 bp, and sonication efficiency was checked on agarose gels. Samples were diluted 1:5 in dilution buffer (0.5 % NP-40, 200 mM NaCl, 50 mM Tris, pH 8) and incubated over night at 4 °C with unspecific control antibody, 2 µl anti-H3K4me3 (17–614 Millipore) or 4 µl anti-H3K27me3 (39535 Active Motif) antibodies. One aliquot, corresponding to 5 % of the input, was stored without antibody treatment. After antibody incubation, the samples were rotated at 4 °C for 3 h with protein A/G Sepharose beads and washed twice in washing buffer (2 mM EDTA, 0.1 % SDS, 0.5 % NP-40, 150 mM NaCl, 20 mM Tris pH 8) and once in final wash buffer (2 mM EDTA, 0.1 % SDS, 0.5 % NP-40, 500 mM NaCl, 20 mM Tris, pH 8). The chromatin was eluted by 2-h incubation and shaking at 65 °C in elution buffer (100 mM NaHCO_3_, 1 % SDS). The genomic DNA was purified using ChIP DNA Clean and Concentrator (Zymo Research) Kit and analyzed by qPCR, to quantify the amount of DNA from the promoter region of selected genes. For data display, the enrichment factor (EF) was calculated from the qPCR threshold cycle values (Ct) relative to input according to the formula: EF (%IP) = 100 * 2^ − [(Ct(5 % IP) − 4.32) − Ct(specific antibody)]. For Figs. 1e and 2a, we wanted to investigate the effects of TSA or VPA in comparison with untreated controls. We compared the ratios of H3K4me3/H3K27me3 of treated cells to the ratios of H3K4me3/H3K27me3 of untreated cells at the respective days of differentiation. Also for transcript levels, the gene expression was presented relative to untreated controls at the respective days. Therefore, H3K4me3/H3K27me3 ratios or gene expression above 1 indicated that TSA or VPA caused an up-regulation compared with untreated control. H3K4me3/H3K27me3 ratios (methylation ratio) or gene expression below 1 indicated that TSA or VPA caused a down-regulation compared with untreated controls. For detailed information on ChIP, primers and antibodies refer to Fig. S11 and S12.

### Statistics and data mining

For statistical analysis of transcript levels and EFs, paired *t* tests were performed using log-transformed expression values relative to hESC, if not stated otherwise in the legend. All data are shown, and all statistics performed refer to biological replicates (=independent experiments).

Over-representation of gene ontologies (GOs) was analyzed using g:profiler (Reimand et al. 2011), with *p* values determined via a hypergeometric distribution. Over-represented GOs were selected, if they belonged to the term domain ‘biological process’ and contained <1,000 genes, and the *p* value was <0.05. For analyses yielding more than 50 GOs, more stringent selection criteria were used: only GOs that had a *p* value <0.001 were selected. For production of the GO word clouds scaling of character size was linearly proportional to the negative logarithm of the *p* value of the respective GO category. GO terms relating to biological processes (bp) were clustered according to their ‘superordinate biological processes’ as described earlier. Example of these larger categories were ‘neuronal differentiation,’ ‘non-neuronal differentiation,’ or ‘migration and adhesion’ (Waldmann et al. 2014).

Venn diagrams were drawn in order to visualize size relations between the compared groups of genes within one diagram. They do not always represent correct ratios, as this would make visualization difficult in case of big size differences. Numbers in Venn diagrams comparing three groups represent the percentage of the (overlapping or unique) part of the diagram relative to samples lysed at DoD4. The corresponding absolute numbers are indicated in the supplementary files. For Venn diagrams with two circles, absolute numbers of PS and their overlap are presented. The numbers that indicate the percentage of the overlap in two group comparisons are relative to the circle that has the same color as the line under the number.

## Results

### Time courses of histone acetylation and altered marker gene expression triggered by HDACi during early neurogenesis

A pure population of neuroepithelial cells (NEP) can be generated from human embryonic stem cells (hESC) within 6 days (Balmer et al. 2012; Chambers et al. 2009; Krug et al. 2013). This model system is characterized by up-regulation of the transcription factors PAX6 and OTX2 (Fig. 1a). We found earlier that continued exposure to TSA and VPA affects the expression of these two genes. In addition, we observed that short drug exposure (for 24 h) showed the expected biochemical effect [increased histone acetylation on day-of-differentiation 1 (DoD1)]. However, when differentiating hESC, treated for the first 24 h with HDACi, were examined on day-of-differentiation 6 (DoD6), there was no effect on the expression of the neuroepithelial markers (PAX6 and OTX2) (Balmer et al. 2012; Chambers et al. 2009; Krug et al. 2013). To better understand the relationship of biochemical changes and the expression of differentiation markers, we started this study by examining the time course of key events including gene expression and various histone modifications.

Analysis of mRNA expression of PAX6 during undisturbed differentiation showed that this gene is very little regulated during the first 3 days, while significant OTX2 up-regulation was already detectable after 2 days. Both genes reached high levels (compared to hESC) on DoD6 (Fig. 1b). Exposure to HDACi (equipotent concentrations of 600 µM VPA or 10 nM TSA) during the entire differentiation process led to a relative down-regulation of both NEP marker genes. Consistent with the time course of developmental up-regulation of the marker genes, the drugs affected OTX2 already from early time points on, while PAX6 levels were only reduced at DoD4–6 (Fig. 1c).

To examine protein acetylation triggered by drug treatment, we used Western blotting. Histone H3 and α-tubulin were selected as abundant and well-characterized target proteins of HDACs. Exposure to TSA or VPA for the first 6 h of differentiation triggered strong acetylation of histone H3 on the whole-cell level. TSA also increased acetylation of α-tubulin (*p* < 0.05), as expected from the HDAC inhibition profile. VPA effects on this target were not significant, which is consistent with the fact that VPA inhibits specifically HDAC class I enzymes and not the tubulin acetylating class II HDACs (Fass et al. 2010; Gottlicher et al. 2001; Khan et al. 2008). Already after 24 h, the extent of protein acetylation was strongly reduced compared with 6-h drug treatment, and after 48 h, the effect vanished (despite the continued presence of the drugs) (Fig. 1d).

This was most likely due to cellular counter-regulations, as HDACs and histone acetyl transferases are dynamically regulated during differentiation (Weng et al. 2012) and adaptive effects have been described for HDACi drug treatment (Kataoka et al. 2013; Tung and Winn 2010). Nevertheless, we also considered that global analysis of protein acetylation may not be sensitive enough. Therefore, we studied histone H3 acetylation of the lysine-27 residue (H3K27Ac) at four promoter sites of interest (transcription start site of PAX6, OTX2, OCT4, Nanog) using chromatin immunoprecipitation (ChIP). No drug-induced changes in the acetylation levels were observed on DoD1 or any of the following days (Fig. S1A). Various control experiments showed that histone acetylation was technically measurable in our cells and that the H3K27 residue can be affected by HDACi (at higher concentrations) at the chosen promoter sites (Fig. S1B). These data suggest that the low, human-relevant developmental toxicant drug concentrations used here triggered at best very weak, non-measurable promoter acetylation. A more persistent effect on gene regulation and differentiation may thus require another histone modification, such as methylation.

### Time course of histone methylation changes triggered by HDACi during early neurogenesis

We had found earlier that a 6-day continued treatment with TSA resulted in alterations of histone methylation, but it remained unclear when such changes happened and how persistent they were (15). As a 4-day treatment with subsequent washout of the drug was sufficient to alter the expression of PAX6 (Balmer et al. 2012; Chambers et al. 2009; Krug et al. 2013), we investigated now in a first pilot experiment, whether the histone methylation pattern was already changed after 4 days and whether such changes would persist throughout a drug washout period. ChIP was performed with antibodies specific for the open/active chromatin mark trimethylated lysine 4 of histone 3 (H3K4me3) and with antibodies specific for the closed/inactive chromatin mark trimethylated lysine 27 of histone 3 (H3K27me3) (Fig. S2). Ratios of H3K4me3 to H3K27me3 enrichment were calculated as a simple measure of the chromatin state [high ratio: open promoter and low ratio: rather silenced promoter (Burney et al. 2013)] (Fig. 1e). As expected, we found the methylation ratio of GAPDH, an actively described gene affected neither by TSA nor by differentiation, to be very high and to remain unchanged. We also confirmed our earlier finding that PAX6 and OTX2 have a high methylation ratio in normal NEP (DoD6) and that TSA strongly reduced this ratio (*p* < 0.001). Now, we found that this effect also held true for DoD4 cells. Moreover, the TSA-induced reduction in the methylation ratio was persistent, when the drug was washed out from DoD4 to DoD6 (*p* < 0.001) (Fig. 1e, Fig. S2). Thus, altered histone methylation patterns correlated with drug effects on neurodifferentiation, and they may play a role in drug-induced developmental toxicity and persistent effects of HDACi.

Therefore, we examined the time course of histone methylations more closely. During normal development, H3K4me3 levels of PAX6 remained relatively constant, while promoter opening was indicated by a decrease in H3K27me3, especially between DoD4 and DoD6 (Fig. 2a). Treatment with TSA reduced this late decrease in the inactivating histone modification (thereby reducing PAX6 transcription) (Fig. 2b, Fig. S3). Opening of the OTX2 promoter during neurodifferentiation was indicated by early (DoD1–2) increases in H3K4me3 and simultaneous decreases in H3K27me3 (Fig. 2a). Upon treatment with TSA, the methylation ratio at the OTX2 promoter was already slightly down-regulated at DoD1 and significantly down-regulated from DoD2 on (*p* < 0.05) (Fig. 2b, Fig. S3). Thus, we found here that prolonged treatment with an HDACi can lead to an enrichment of inactivating histone modifications. This offers a mechanistic explanation for the down-regulation of important developmental genes (here PAX6 and OTX2) by TSA. These findings differ largely from those reported on short treatment (Boudadi et al. 2013; Jergil et al. 2011; Nightingale et al. 2007), which increases the amount of chromatin-opening histone modifications. On this basis, it became highly interesting to obtain more data on the persistence of altered histone methylations upon pulsed drug treatment.

### Effects of pulsed drug treatment on histone methylation and NEP differentiation

After we had found that the increase in H3K27me3 as well as the down-regulation of PAX6 and OTX2 was persistent when the cells were treated for 4 days, followed by a 2-day wash out (Fig. 1e), we investigated the minimum treatment period required to induce this stable effects.

The cells were exposed to TSA for 1, 2, or 3 days, before the drug was removed, and differentiation was continued until DoD6. These pulsed treatments (P1d, P2d, and P3d) were compared to continuous exposure to TSA (Fig. 2c). As controls of the phenotypic effect of disturbed differentiation, we quantified the protein levels of PAX6 and OTX2. Short treatments of up to 2 days had no effect, while longer drug treatment resulted in strongly decreased levels of the phenotype markers of NEP (Fig. 2d). Thus, drug treatment of about 3 days was sufficient to cause developmental disturbances.

In parallel, we examined how the pulsed treatment affected the gene expression and promoter methylation of the NEP marker genes. As seen for the protein, 3-day treatment was sufficient to cause the same extent of down-regulation as continuous drug exposure (Fig. 2e). Examination of H3K4me3 and H3K27me3 marks showed that 3-day drug treatment, followed by 3-day washout triggered at least the same extent of change (i.e., increase in H3K27me3), as continuous drug treatment. In the case of OTX2, about 50 % of these changes were already triggered by 1–2-day treatment, while changes in the PAX6 promoter required up to 3 days of drug exposure (Fig. 2f). Under this set of experimental conditions, the developmental disturbance triggered by different drug exposures correlated well with altered methylation patterns. The data suggested that a 3–4-day exposure should definitely be sufficient to alter the differentiation track of the cells and to affect gene expression independent of the continued presence of drugs. However, an altered expression of only two NEP markers is not sufficient to answer the question, if we observe really a changed differentiation track. To explore this, we switched from the analysis of few stage-specific markers to full transcriptome analysis of the treated cells.

### Transcriptome changes triggered by exposure to HDACi for various time periods

**Fig. 3:**
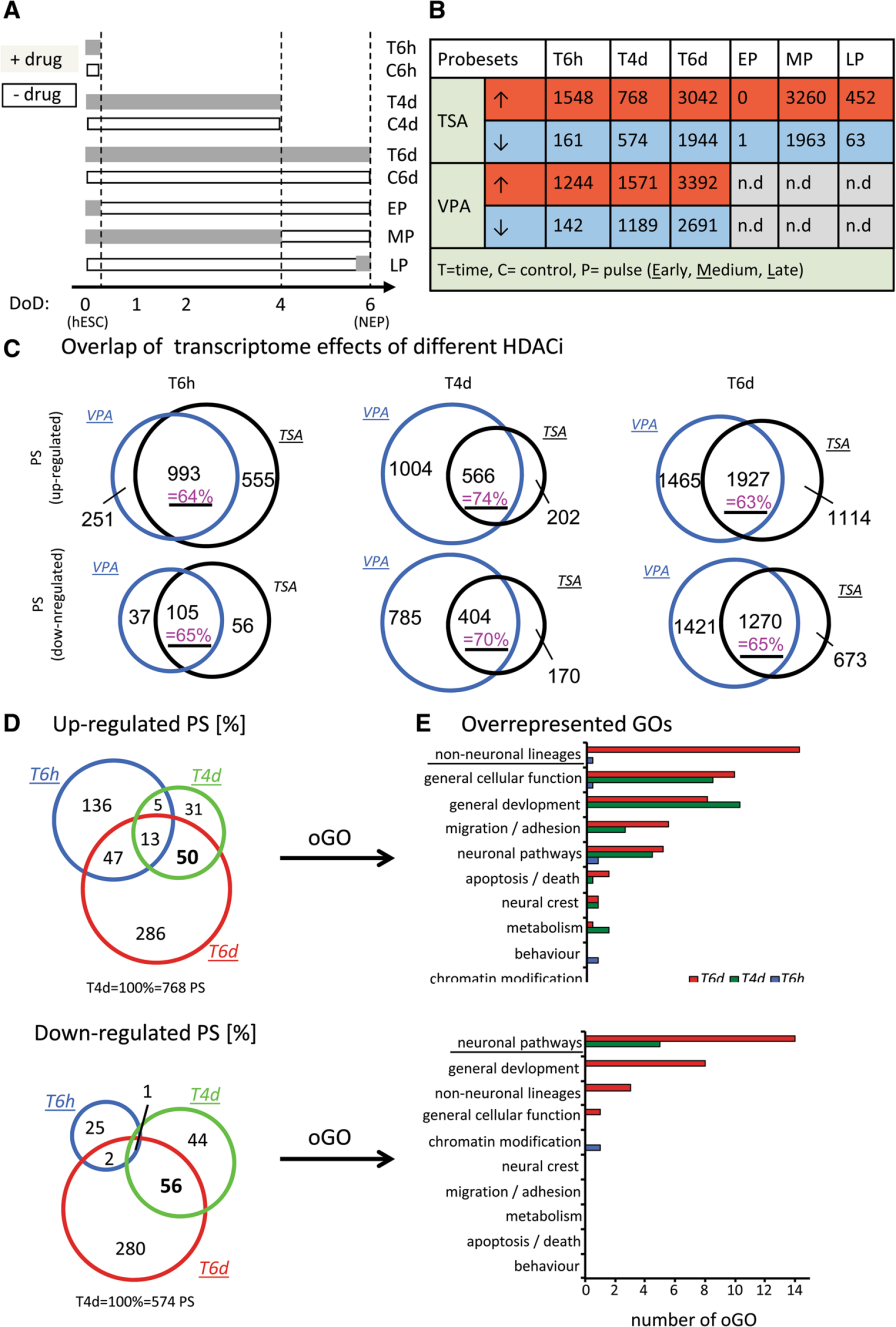
Transcriptome analysis after different treatments with TSA and VPA. **a** Overview of the different exposure scenarios during differentiation. *Bars* indicate the duration of differentiation before samples were taken, and *gray shading* indicates the period of drug exposure within that time. **b** Differentiating cells were treated as indicated in **a**; mRNA was prepared and analyzed by Affymetric DNA microarrays. The numbers of probe sets (PS) regulated upwards (*red*) or downwards (*blue*) in the presence of TSA (10 nM) or VPA (600 µM) are given in the table. **c** Venn diagrams display absolute numbers of regulated PS (*p* < 0.05, FC > 1.5 or FC < 0.67) induced by TSA, VPA, or both during the indicated treatment periods. The percentage of overlap of the drug treatments is indicated in *purple*. **d** Venn diagrams display PS altered by TSA treatment (*p* < 0.05, FC > 1.5 or FC < 0.67) for different time periods. The *numbers* in the sectors are given as percentages of the number at T4d (absolute numbers are in supplements). **e** Overrepresented gene ontology terms (oGOs) were determined from the regulated PS indicated in D. Individual oGOs were classified by their assigned to superordinate cell biological processes, and the number of oGO for each of these (e.g., migration or neuronal pathways) is displayed. Complete data sets on regulated PS, oGO, and superordinate biological processes are given in Tables S2 and S3 (color figure online)

To obtain baseline information on the cell differentiation, the temporal alterations of the transcriptome were recorded first for undisturbed cells. Samples were taken after 6 h, DoD4, and DoD6, and they were related to the expression in hESC (Fig. 3a, C6h, C4d, C6d). Gene expression levels were measured on DNA microarrays for four complete and independent time course experiments. Principal component analysis (PCA) of the data sets showed that the differentiation model was robust, and large numbers of probe sets (PS) changed reproducibly over time (Fig. S4). All PS significantly regulated at any time point relative to hESC (*p* value <0.01, fold change (FC) > 1.5 or FC < 0.67) were identified (Fig. S4B, C, Tab. S1). Neuronal development and maturation were the most conspicuous themes within the up-regulated PS on DoD6 (Fig. S4D). The overrepresented gene ontology terms (oGOs) in down-regulated PS gave mainly evidence of the known (Xu et al. 2013) metabolic changes related to mitochondrial and amino acid metabolism (Fig. S4D). Altogether, these background data confirmed the large-scale changes, which the transcriptome has to undergo for correct lineage commitment, although only few PS (5 %) were found to be regulated at all time points (Fig. S4B, C) (Burney et al. 2013; Chambers et al. 2009; Coskun et al. 2012).

Having established baseline model behavior, the time-dependent effects of TSA and VPA on transcriptome changes were examined (Fig. 3a, T6h, T4d, T6d). Early exposure for as little as 6 h (T6h) was sufficient to up-regulate >1,200 PS; but only about 150 PS were down-regulated. All these early regulations were reversed after a 6-day washout period (EP). Treatment for 4 days (T4d) resulted in similar numbers of up-/down-regulated PS (total: 1,342 PS for TSA, 2,760 PS for VPA). About 5,000–6,000 PS were regulated when cells were exposed for 6 days (T6d) (Fig. 3b, Tab. S2). Bootstrapping outlier analysis showed that the smaller number of PS at T4d (compared to day 6) was not due to a particularly high experimental variance of the samples (Fig. S5).

TSA and VPA are HDACi with a >10,000-fold difference in potency and with little structural similarity. However, 62–74 % of the PS regulated by TSA were also altered by VPA (Fig. 3c). The high degree of overlap at all time points strongly suggests a common mode of action (HDAC inhibition) of both compounds. The analysis of potential transcription factor binding sites in the regulated PS for each compound showed >90 % overlap (not shown) and further confirmed a similarity of the MoA. As TSA is the more specific drug, we focused further work strongly on this compound.

As we aimed to explore whether we indeed induce a wrong differentiation track with long treatments, but not with short treatments, we next compared the differentially expressed PS that are induced by short, medium, and long treatment with TSA. Comparison of the PS affected by different exposure periods of TSA showed that the overlap was only very small (1–13 %). An important implication of this is that the data obtained after 6 days of exposure did not reflect the initial/direct response to drug exposure (Fig. 3d, Fig. S6). In addition, the data showed that the final outcome of such toxicogenomics experiments in a developing cell system depended to an astonishingly high degree on the time point of analysis (e.g., T4d vs T6d). To obtain an overview of the biological implications of the drug-induced transcriptome changes, we used two levels of data clustering. First, oGOs were determined among the up- and down-regulated PS. Then, the oGOs were binned according to related biological processes, such as ‘apoptosis’ or ‘metabolism’ (Waldmann et al. 2014). From this, a clear picture emerged: the down-regulated genes for T6d mainly reflect down-regulation of neuronal pathways and general development. This fully corroborated the neurodevelopmental toxicity character of the model. The up-regulated genes strongly indicated differentiation to non-neuronal lineages (Fig. 3e, Tab. S3). Therefore, we conclude that long- but not short-term treatment indeed changes the gene expression pattern toward a wrong (non neuronal) differentiation track.

The divergence from neuronal differentiation to non-neuronal lineages (according to oGOs) developed very strongly between T4d and T6d, i.e., during the time, when drug treatment was not important anymore for neurodevelopmental marker expression. From this and from our initial experiments (Figs. 1, 2), we concluded that overall transcriptome changes should be similar for T6d treatment and a medium drug pulse of 4-day exposure followed by a 2-day washout (MP).

### Transcriptome changes gain independence of drug presence after 4 days

**Fig. 4:**
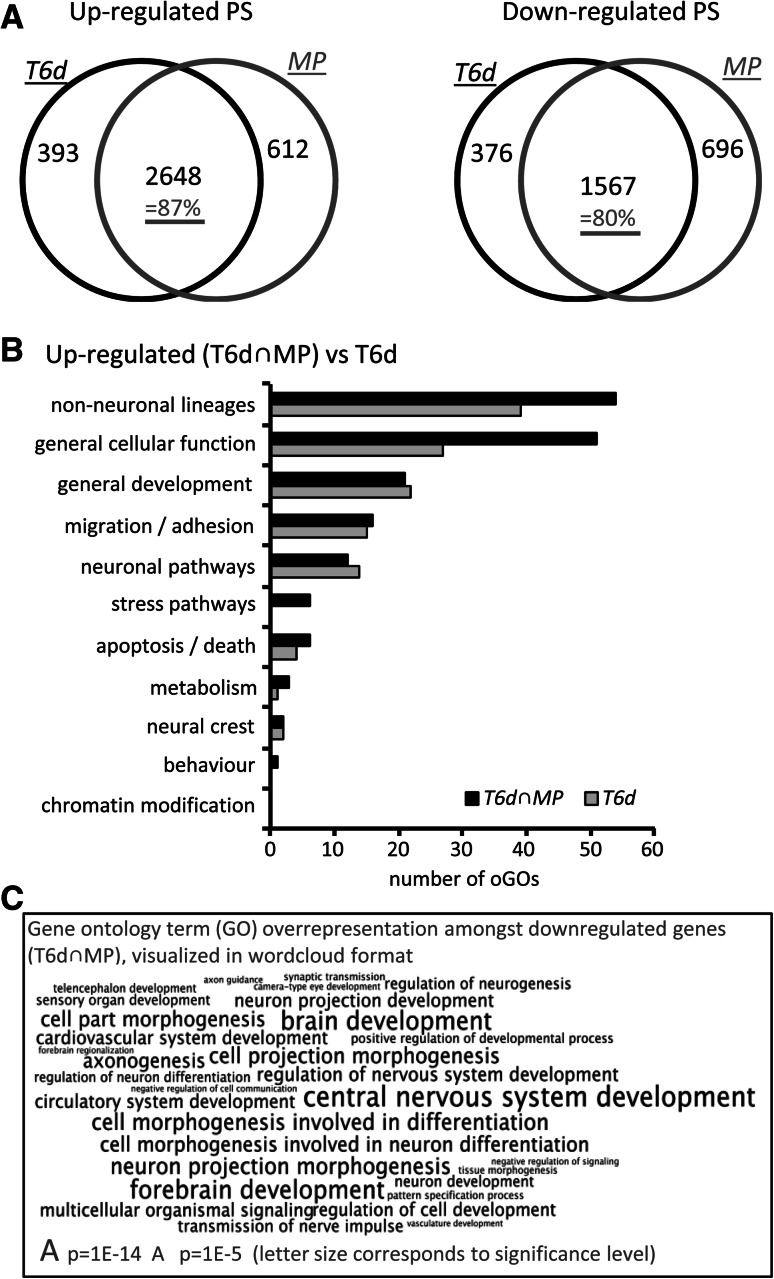
Concordance of transcript changes after continued treatment or drug washout on DoD4. **a** Cells were differentiated in the presence of TSA for 6 days (T6d) or for 4 days followed by a drug washout period of 2 days (medium pulse, MP) and analyzed by Affymetrix DNA microarrays. The absolute numbers of up- and down-regulated PS are indicated in Venn diagrams. The percentage of overlap is indicated in *purple*. **b** The oGOs were determined from the up-regulated PS of T6D treatment as well as from the overlap of T6D with MP (T6d ∩ MP). The oGOs were further classified into superordinate cell biological processes, and the numbers in these categories are displayed. For full information on regulated PS, oGO, and superordinate biological processes, see Tables S2 and S4. **c** Significantly down-regulated PS were determined for T6d ∩ MP. GOs overrepresented among the PS down-regulated are displayed as word cloud. The character size is scaled according to the *p* value of the corresponding GO (color figure online)

We addressed the question of continued transcriptome effects in the absence of drugs by comparing T6d and MP (Tab. S2). The regulated PS showed a very high overlap of 80–87 % between MP and T6d (Fig. 4a, Fig. S7). The oGOs among up-regulated PS pointed to the differentiation of several other cellular lineages (Fig. 4b, Tab. S4), such as the cardiovascular system, neural crest, skeletal system, and glands. Moreover, there was a high overlap of oGOs among T6d PS and the T6d/MP overlap PS (Fig. 4b). The higher GO enrichment among the latter set of genes was most likely due to the lower percentage of non-specific genes, eliminated through the overlap filter. The commonly down-regulated PS gave overwhelming evidence of disturbed neurodifferentiation (according to oGOs) (Fig. 4c, Tab. S4). We wondered whether the biological response to the drugs could have been predicted already on day 4. At this time (T4d), the majority [64 % (up)/55 % (down)] of TSA-regulated PS was the same as found after 6 days (Fig. S7A). The PS of T4d that overlapped with T6d and MP pointed already to disturbed differentiation: the oGOs among these down-regulated PS indicated a defect in forebrain development (Fig. S7B) and the up-regulated PS pointed again to an increase in unwanted differentiation tracks (Tab. S4). In summary, drug treatment for 4 days was sufficient to trigger the definite deviation from the normal differentiation path. From this point on, differentiation of wrong lineages most likely contributed to further increases in transcriptome changes (compared to control cells). This wrong differentiation track continued to deviate more and more from control cells, also in the absence of the drugs.

### Comparison of continued drug exposure and short exposure of NEP

**Fig. 5:**
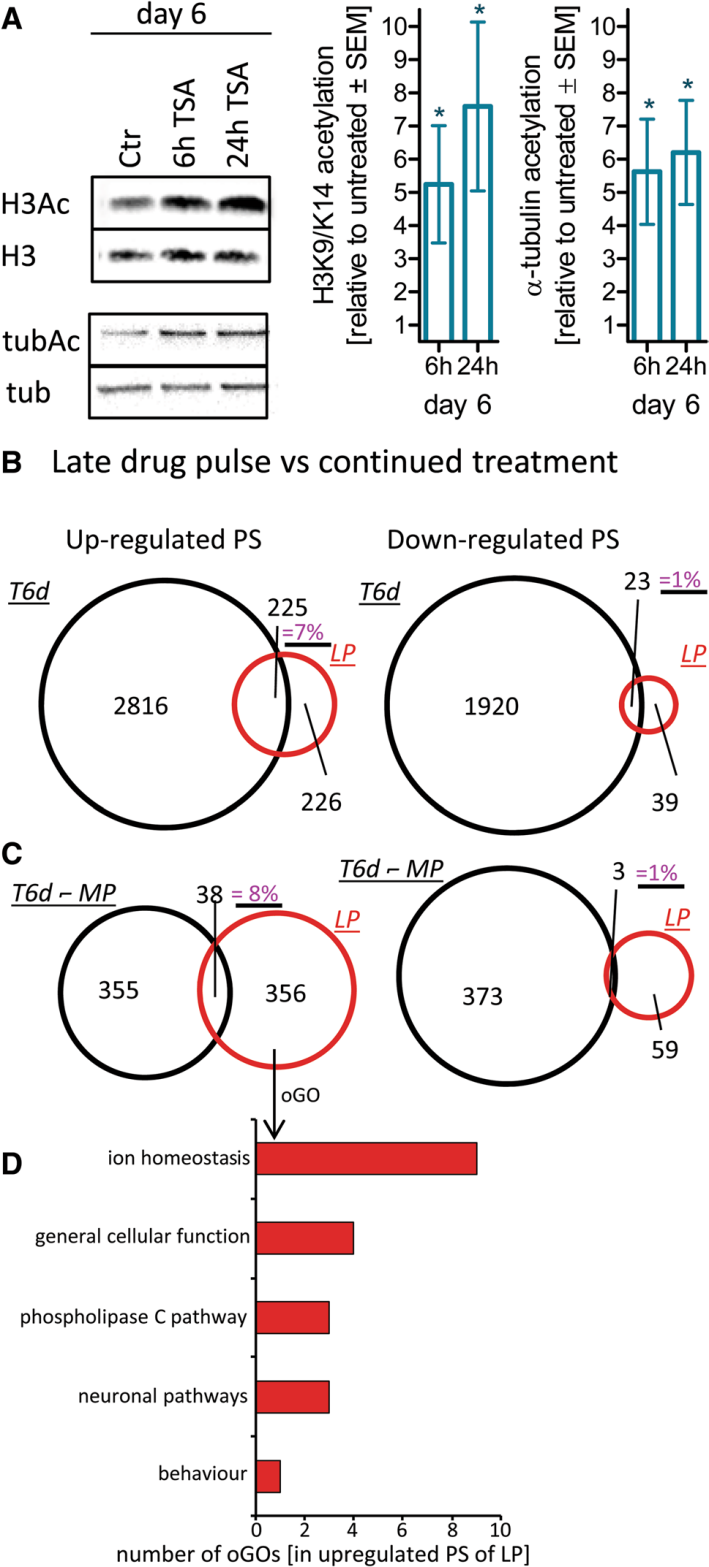
Comparison of the acute and chronic (long-term treatment) effects of TSA. **a** Cells (hESC) were differentiated for 6 days to NEP, and TSA (10 nM) was added only during the last 6 h or 24 h. Then, protein acetylation was determined by Western blot as in Fig. 1. Data are mean ± SEM of three experiments. *p < 0.05. **b** Venn diagrams display the number of PS altered by drug treatment (*p* < 0.05, FC > 1.5 or FC < 0.67) after continuous (T6d, *black circle*) exposure, or after late, pulsed (LP) treatment (last 6 h of the 6-day differentiation, *red circles*). **c** Venn diagrams compare PS triggered by a late drug pulse (LP, *red circles*) with PS regulated by continuous drug exposure, but not found under MP washout conditions (T6d without—MP, *black circles*). The percentage of overlap is indicated in purple. **d** The GOs overrepresented among up-regulated PS of LP incubations were classified into superordinate cell biological processes (full information on regulated PS, oGO, and superordinate biological processes: Tables S2 and S4) (color figure online)

The independence of the transcriptome response from the presence of drugs suggested that the transcriptome changes did not inform on direct drug signaling. To address how the cells would react to short drug exposure, we generated NEP and exposed them to TSA only during the last 6 or 24 h of the 6-day differentiation. This treatment caused a pronounced increase in histone acetylation and tubulin acetylation (about fivefold to eightfold, *p* < 0.05) (Fig. 5a). These results showed that HDACi could trigger the expected biochemical response (acetylation) in NEP acutely exposed to the drugs. This effect was similar in size as the one in cells pulsed with drug during the first 6 h of differentiation (Fig. 1d). A further similarity was that also the late drug pulse (LP) triggered a pronounced transcriptome response within 6 h. However, the regulated PS differed strongly from those of the T6d data set (Fig. 5b). Only 7 % of the up-regulated T6d PS and only 1 % of the down-regulated ones were contained in LP (Fig. 5b). It may be argued that T6d contains two types of regulated genes: PS altered because of an overall altered differentiation track and PS altered because of the continued presence of the drug. The latter set of genes may be termed ‘affected by continued drug presence after long-term exposure.’ To get information on this set of genes, we subtracted the MP (washout) PS from the T6d PS. This left 393 up-regulated PS and 376 down-regulated PS. Even this subset of genes (that should in theory be enriched for PS affected by the presence of TSA) overlapped only 8 % (up) or 1 % (down) with the LP PS (Fig. 5c). These findings only corroborated our earlier conclusion that the PS of T6d or MP hardly reflected any direct responses to drug exposure.

The direct drug response (LP), related to the change in acetylation, was further characterized by analysis of GO overrepresentation. This pointed to the biological processes of ‘ion homeostasis’ and ‘phospholipase C signaling,’ and they differed strongly from the long-term response (Fig. 5d, Tab. S3).

### Differences and similarities of transcriptome changes associated with early direct drug response versus continued long exposure

**Fig. 6:**
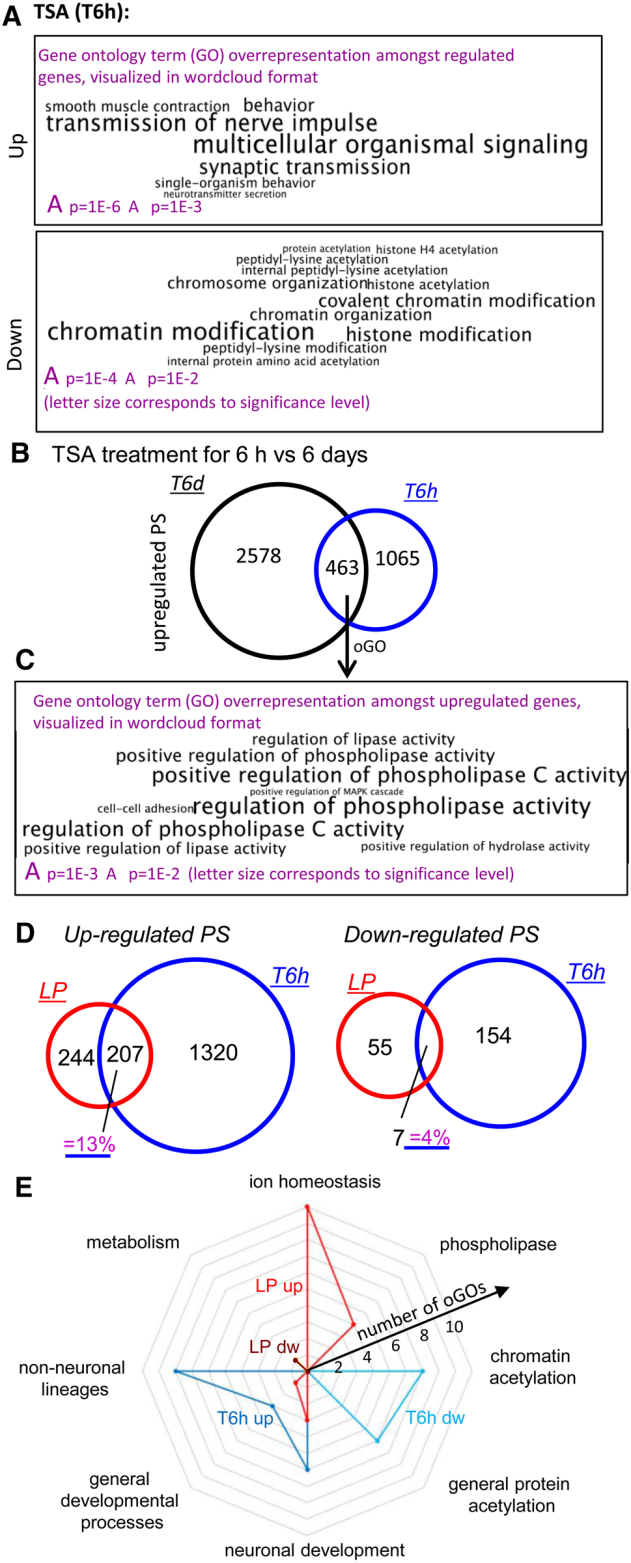
Differences of acute effects of TSA either early or late during NEP differentiation. **a** Overrepresented GOs were determined from significantly up- (*upper panel*) and down-regulated (*lower panel*) PS from T6h samples. They were displayed as word clouds with character size scaling according to the *p* value of the corresponding GO. For full information, see Table S2 and S3. **b** Venn diagrams compare numbers of up-regulated PS of long-term (T6d, *black circle*) and early short-term (T6h, *blue circle*) treatment with TSA. **c**. GOs overrepresented among the PS of the overlap shown in **b** are displayed as a word cloud. **d** Venn diagrams compare numbers of regulated PS of short-term TSA treatments at early (T6h, *blue circle*) and late (LP, *red circle*) time points. The percentage of overlap is displayed in *purple* (**e**). The oGOs determined from significantly up-regulated (*blue*) or down-regulated (*red*) PS of the LP and T6h incubations were assigned to superordinate cell biological processes. These were then used as axes on a radar plot, with the distance from the center indicating the number of oGO on each axis. For full information on regulated PS, oGO, and superordinate biological processes, see Tables S2 and S3 (color figure online)

We explored whether the immediate drug response to TSA at the beginning of the experiment (differentiating hESC exposed during the first 6 h = T6h) was sufficiently predicting T6d. Analysis of oGO among T6h up-regulated PS pointed to altered signaling/nerve activity, and developmental GOs were not overrepresented at all among up-regulated PS (in contrast to T6d). The PS down-regulated by TSA were indicative of one major underlying biological process: Six of the 13 oGOs were related to chromatin modification and 7 to acetylation (Fig. 6a, Tab. S3). A similar response was observed for VPA (Fig. S8), and these findings are well consistent with major changes in histone acetylation. Both HDACi affected genes related to chromatin modification, such as ING5, KAT5 (histone acetylation), and several PHF genes (involved in chromatin remodeling and transcriptional regulation). Thus, one primary effect of VPA and TSA was alteration of genes involved in chromatin structure (Fig. 6a, Fig. S8). Such epigenetic mechanisms triggered early by HDACi, and fixed by longer presence of the drugs, could initialize the massive changes observed later after prolonged drug treatment.

A direct comparison of the T6h and T6d transcriptome changes showed that 70 % of the PS up-regulated early were not up-regulated late (T6d) (Fig. 6b), and 90 % of PS down-regulated early were not down-regulated late. These findings were similar for TSA and VPA (Fig. S9). Only 13 of 303 oGOs among up-regulated T6d genes (*p* < 0.05) overlapped with oGOs from up-regulated T6h genes (Fig. S10). These data indicate very clearly that the early drug response predicted the overall outcome after 6 days only poorly, and vice versa the primary effects of TSA may not be identifiable from measurements at late time points.

However, there were at least some similarities of the responses: 463 PS overlapped for TSA responses after 6 h and 6 days (Fig. 6b). All oGOs among them pointed to altered signaling events, mainly related to phospholipase C (Fig. 6c, Table S4) as possibly common response feature. To narrow down the common drug responses to effects specific for HDACi, we extracted PS that were regulated by both TSA and VPA and at both time points (Fig. S9A). Eleven GOs were overrepresented among these PS (Fig. S9B), with 7 of them referring to phospholipase C regulation. They involved, e.g., EGFR, KIT, PDFGRA, and EDNRA. The other four oGOs referred either to neural crest (NC) or to their guidance and migration (comprising TWIST1, SOX10, SNAI2, and SEMA3C) (Zimmer et al. 2012). Thus, the PS up-regulated by HDACi both early and late suggested that NC is one of the cell lineages that was erroneously generated in our model when differentiation was disturbed by drugs.

### Dependence of immediate transcriptome responses to HDACi on the developmental stage

The above findings suggest that longer exposure is not suitable for defining the mode of action of drugs. Some literature data suggest that short drug pulses of few hours may be more suited (Jergil et al. 2011; Theunissen et al. 2012). However, this raises the question, when such short-term exposure should be performed. A developing biological system does not only consist of one defined cell type, but it rather reflects all biological stages between the starting point and the final population. On this basis, we hypothesized that the ‘cellular’ response, i.e., short-term transcriptome alterations would strongly depend on the time point of measurement, i.e., on the differentiation stage. To test this, we compared the responses to 6-h TSA treatment of cells at the early stage of differentiation (T6h) and at the final stage (LP). The down-regulated PS of the two conditions showed hardly any overlap at all. For the up-regulated PS, 13 % of the T6h PS were also found in LP and 46 % of the PS of LP were found in T6h (Fig. 6d). GOs overrepresented in commonly up-regulated PS referred only to regulation of phospholipase activity. Besides this hint toward signal transduction being affected in both cases, there was little further indication of common functional effects. On the contrary, the representation of superordinate biological processes by the oGOs was largely divergent between the two 6-h pulse experiments (Fig. 6e, Tab. S3). We conclude from this that typical HDACi target genes that inform on the mode of action do not exist as such. They depended to a very high degree on the experimental setup, the time point of measurement, as shown here, and they also depended to a high extent on the length of exposure, as shown in the above paragraphs.

## Discussion

Transcriptomics studies have been instrumental for characterizing the development of model organisms, the human brain, or various stem cells (Kang et al. 2011; Mariani et al. 2012; Xie et al. 2013; Yang et al. 2013). Most available data refer to fixed time points, but some studies already showed the highly dynamic behavior of such systems (Theunissen et al. 2012; Zimmer et al. 2011). An additional layer of complexity is added, when experimental disturbances are studied in systems that change over time. A stressor that switches a static system from one state to an other one would be expected to shift such a dynamic system from one developmental track (dynamic series of changes) to another. Second and third levels of additional complexity are added, if the type and extent of such shifts depend on the length of exposure and on the time point of exposure relative to the normal differentiation track. Although the understanding of the temporal evolution of a developing biological model system under stress is essential in toxicology and pathophysiology, we are not aware of any study that has addressed this issue in a quantitative and systems-wide way. Three major conclusions from our study can form a basis for further exploration of this field:

First, it became clear that the usual (long-term exposure) toxicogenomics data recorded from developing stem cells describe to a large extent the cellular phenotypic changes and only to a small extent the direct drug action as such. Second, drug actions in a dynamic stem cell system can be fully reversible, even when the expression of hundreds of genes has been changed. However, at a certain point, permanent and persistent changes are triggered that can become independent of the continued presence of drug. We found that chromatin alterations in particular histone methylation could act as persistence detector or irreversibility switch. Third, even the short-term immediate effects on the transcriptome depended on the time point of drugs application. This apparently trivial finding, considering the dynamic behavior of a differentiation system, has important implications for future systems toxicology studies: Exposures at multiple times need to be performed in order to obtain data that sufficiently describe drug effects and responses of the cells.

Do transcriptomics data reveal the mechanisms by which a compound interferes with neural development? (Theunissen et al. 2012). Our data suggest unambiguously that final changes in the transcriptome at the end of the differentiation period mainly define the cellular phenotype. This is supported by the following six lines of evidence. (a) Primary drug responses to HDACi are dominated by up-regulated genes (Berger 2007). The chromatin opening due to acetylation may be enhanced and then stabilized by increased H3K4me3 (Nightingale et al. 2007). We find here equal numbers of up- and down-regulated PS for the T6d condition. This corroborates findings in the literature (Krug et al. 2013; Theunissen et al. 2012) and suggests that indirect responses play a large role after longer (>6 h) incubation periods. (b) The primary biochemical drug response (acetylation) was not detectable after prolonged exposure. Thus, it is unlikely that the transcriptome response at late time points was triggered by a direct response to HDACi. (c) The long-term (T6d) response differed considerably from the acute response at the same time (LP). About 95 % of the T6d PS were not predicted by the LP direct drug response data. (d) The transcriptome response after a 2-day washout of the drugs (MP) was very similar to the response without washout (T6d). This makes it very unlikely that a direct drug response was measured at T6d. (e) Moreover, it should be assumed that the transcriptome response data should be to some degree independent of the experimental system, if they would characterize the MoA of drugs. However, we observed that closely related hESC-based systems showed very different responses to VPA (Krug et al. 2013) and apparent HDACi consensus genes (Jergil et al. 2009, 2011) defined in murine cells by overlapping responses of different drugs were not regulated in our cells. In summary, this corroborates our hypothesis that transcriptome data from disturbed developmental systems reflect mainly an altered phenotype and that information on direct drug effects requires short drug treatments of up to 6 h. (f) When the overlap of drug-affected ‘toxicant-response’ genes (T-genes) and developmentally regulated gene (D-gene) clusters was examined, we found that 90 % of the genes regulated late during differentiation were affected by HDACi. This high percentage of overlap is hard to explain by primary drug action; in particular as TSA acted early, and the changes occurring between day 4 and day 6 did not even require the presence of the drug. Instead, the observation is plausible, if it is assumed that the drugs changed the overall development, and therefore nearly all later phase D-genes were affected. Thus, the apparently specific effects of HDACi on neurodevelopment may be mainly due to the fact that the system studied was based on neurodevelopment. One may then hypothesize that HDACi may preferentially target cardiac genes in a cardiac development model. Such findings have indeed been obtained from differentiating mESC. VPA affected their neural development upon neurodifferentiation and cardiac development upon cardiac differentiation (Theunissen et al. 2013).

The effects of a toxicant may depend not only on the time point of measurement, but also on the duration of exposure. Some developmental toxicity responses may require the activation of a ‘persistence detector’ to distinguish between short reversible interactions on the one hand and toxicity on the other hand (Lim et al. 2013). For instance, epigenetic changes have been suggested as persistence switches for ethanol sensitization (Botia et al. 2012; Qiang et al. 2011). An altered state of cells is also fixed by so-called ‘gateway drugs.’ These are compounds allowing the later action of other drugs, even when they have been washed out for a long time. This is particularly important in the field of addiction, and the mode of action has also been explained by histone modifications (Levine et al. 2011).

Molecular persistence mechanisms are of high importance in the field of developmental toxicity, in which compounds might show effects years after the exposure has taken place (Kadereit et al. 2012). Evidence is emerging that altered behavior and late-onset disorders that are triggered early in life are associated with epigenetic alterations (Rudenko and Tsai 2013; Weaver et al. 2004). Early developmental exposure to toxicants could result in an accumulation of epigenetic changes that, when reaching a certain threshold, result in transcriptome alterations associated with adverse health effects. There are several examples for toxicants or stressors that can trigger diseases in later life when exposure takes place in utero or childhood or that can even trigger trans-generational effects (McGowan et al. 2009). Early exposure to lead has, for example, been associated with Alzheimer’s disease (Wu et al. 2008).

**Fig. 7:**
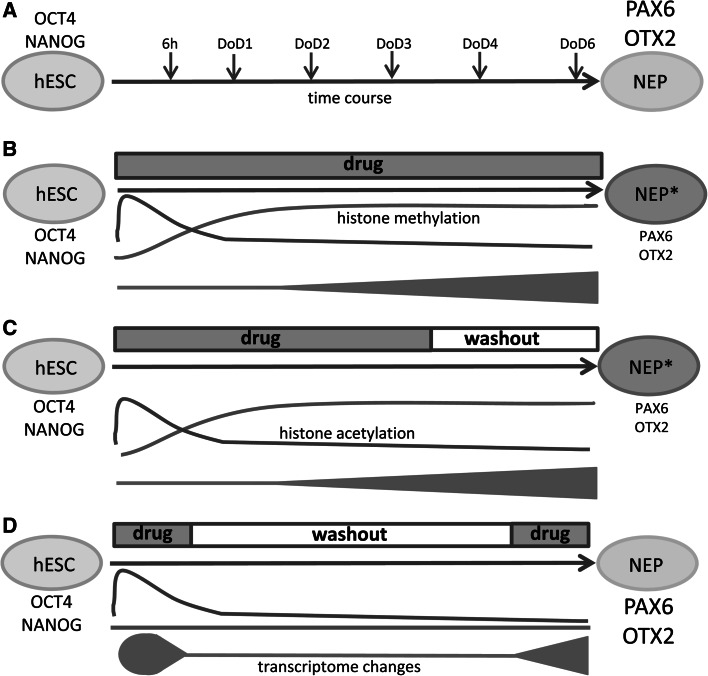
Summary. **a** Neuroepithelial precursors (NEP) were generated from hESC within 6 days of differentiation (DoD). Main cell type markers are indicated. **b** Continuous drug treatment led to an altered NEP differentiation (NEP*) as indicated by transcriptome changes (*blue*), reduced marker expression (PAX6, OTX2), and permanent changes in histone methylation (after 2–3 days) at the promoters of the markers. Acetylation changes were only transient. **c** Drug exposure for 4 days resulted in the same disturbed NEP differentiation as continued drug exposure. The number of altered PS and the alterations of histone methylation were not affected by drug washout. **d** Short-term drug treatment induced a strong, but fully reversible change in gene expression. Methylation of marker promoters was not altered, and marker expression was normal. The transition of transient drug-induced gene expression and histone acetylation changes to a permanently altered transcriptome and NEP differentiation correlated with the permanence of promoter histone methylations (color figure online)

The model system chosen here was based on the known neurodevelopmental disturbances of HDACi and the associated transcriptome responses (Fig. 7a, b). We used this as a test case to study time-dependent transcriptome responses. A short pulse of TSA (EP) triggered a strong transcriptional response that was fully reversible, while a longer exposure (MP) triggered a persistent effect, even after discontinuation of drug exposure. The transient response correlated well with acetylation. The persistent response did not correlate with histone acetylation, but rather with a shift of the histone methylation ratio (Fig. 7c, d). This shift required more time and was specific for the gene studied. For instance, OTX2 was affected earlier than PAX6, and the direction of methylation changes in the OCT4 promoter (Balmer et al. 2012) was opposite to the one described here for PAX6. Histone methylations are well suited for deciding on cell fate and long-term regulations, as they can favor transcription (H3K4me) or attenuate transcription (H3K27me), depending on the site of modification. Histone methylations do not usually change globally (i.e., their overall cellular level remains constant), but the pattern of the different methylases and demethylases can change dramatically during early neural differentiation (Weng et al. 2012), and individual promoters are affected in a highly specific manner. In particular, the ratio of promoter H3K4/H3K27 trimethylation has been shown to reflect the phenotypic plasticity of stem cells during neural fate decision. Changes in this ratio at promoters of genes associated with neural differentiation frequently precede the changes in gene expression (Burney et al. 2013).

If it is assumed that cells can react reversibly to short stimuli, as shown here, and that the duration of exposure plays a role for the overall outcome, then a mechanism functioning as drug persistence detector is required. The easiest way of imagining such a mechanism could be a superordinate regulator of a wrong pathway (e.g., a neural crest organizer) whose promoter chromatin structure functions as Boolean AND element being switched by acetylation *plus* a second alteration that takes more time in response to drug exposure. Short drug exposure would only trigger acetylation and thus not be sufficient for activation/switching. Longer exposure would still allow for acetylation and now also for the second change. Together, they would lead to activation of the superordinate regulator, and the activation state would be fixed by the histone methylation pattern. At present, this is a speculative hypothesis just intended to give an idea how underlying regulations may be imagined; the model system chosen may be too complex to provide causal evidence for such a mechanism with the technology presently available. Further studies will require considerable technical optimizations. ChIP can easily be accommodated to a whole genome level by applying sequencing instead of PCR as endpoint. Yet, the real issue lies in the quantification across several independent experiments with human stem cells, and the handling of the type of information resulting from this. Quantitative detection of compound-induced histone changes requires a high level of robustness and sensitivity of the method, and this is much harder to achieve for ChIP than for methods such as PCR, DNA microarray, or Western blot. Already in the present study, considerable method optimization was required to obtain reproducible and statistically significant data on the chromatin changes taking place at few selected marker genes.

The acute responses to HDACi differed significantly from the response to continued treatment. More importantly, they differed also from one another. No matter whether the experiment was performed at the beginning or toward the end of differentiation, a vastly larger number of genes were up-regulated than down-regulated (as expected of HDACi). But different genes were affected. The 6-h time point has been found to be optimal in previous studies, e.g., on stem cells (Jergil et al. 2011) or a large number of human tumors (Cohen et al. 2011) to record direct drug effects. We conclude that the direct drug effect was different at distinct times of differentiation. This corroborates earlier findings of VPA acute cytotoxicity being highly dependent on the developmental stage (Fujiki et al. 2013). Taken together, these findings imply that a potential mode of action of a developmental toxicant is not an intrinsic drug property, but it is a combined feature of the experimental system and the chemical used. In this respect, developmental toxicity may differ from other fields. Its testing may therefore require a battery of parallel tests and particularly complex systems toxicology approaches (Hermsen et al. 2013; Tonk et al. 2013) relative to more acute forms of toxicity. It needs to be established whether approaches, as in the ToxCast program (Sipes et al. 2011, 2013), that rely mainly on hundreds of simple assays for biochemical/cellular targets can help to substitute or to complement the complex differentiation models used in a test battery (Piersma et al. 2013).

Our study has major implications for the design and interpretation of toxicogenomics data in development. We found that classical transcriptome data from a disturbed/stressed differentiation model strongly reflect the altered phenotype. In the case of HDACi, the phenotype contribution is >90 %. This number may be smaller or larger for other stressors, but it will most likely always be sizeable. The question arises what this transcriptome information can be used for, if it does not inform on the mode of action of a chemical. We assume that different types of stressors result in different developmental disturbances and therefore also different transcriptome patterns (Balmer et al. 2012; Krug et al. 2013). Thus, the information will be useful for compound classification, differentiation, and possibly potency ranking (Schulpen et al. 2014). Beyond this, there is a further dimension of information contained in the transcriptome data. If they are largely independent of direct compound effects, then they constitute a comprehensive phenotypic description of the culture state. In the classical toxicological literature, it has always been assumed that transcriptome endpoints require phenotypic anchoring to other types of endpoints (Paules 2003; Waters and Fostel 2004). Our data suggest now that the comprehensive transcriptome data can be a phenotypic anchor as such. Transcriptome data may be more comprehensive and robust than classical endpoints (such as immunostains). It appears worthwhile to develop quantification tools that indicate, on the basis of the transcriptome as phenotypic descriptor, how big a developmental insult is, and that allow the ranking of unknown drugs, or of different concentrations or exposure times of one given drug (Waldmann et al. 2014).

## Electronic supplementary material

Below is the link to the electronic supplementary material.

Supplementary material 1 (PDF 921 kb)

Supplementary material 2 (XLSX 928 kb)

Supplementary material 3 (XLSX 1069 kb)

Supplementary material 4 (XLSX 92 kb)

Supplementary material 5 (XLSX 109 kb)
