# Supplementary material for: From transient transcriptome responses to disturbed neurodevelopment: role of histone acetylation and methylation as epigenetic switch between reversible and irreversible drug effects
Source: Arch Toxicol. 2014 Jun 17;88(7):1451–68. doi: 10.1007/s00204-014-1279-6 (PMC4067541; doi:10.1007/s00204-014-1279-6)
Supplement: Supplementary file 1 — Supplementary material 1 (PDF 921 kb) [file 204_2014_1279_MOESM1_ESM.pdf]

Supplemental Material for

From transient transcriptome responses to disturbed neurodevelopment: role of histone acetylation and methylation as epigenetic switch between reversible and irreversible drug effects

Nina V. Balmer, Stefanie Klima, Eugen Rempel, Violeta N. Ivanova, Raivo Kolde, Matthias K. Weng, Kesavan Meganathan, Margit Henri, Agapios Sachinidis, Michael R. Berthold, Jan G. Hengstler, Jörg Rahnenführer,Tanja Waldmann and Marcel Leist

Table of contents

Figure S1: Experimental controls for histone acetylation analysis

Figure S2: Details on histone methylation changes as basis for Fig. 1E.

Figure S3: Details on histone methylation changes as basis for Fig. 2A.

Figure S4: Transcriptome changes during early neuroectodermal differentiation.

Figure S5: Quality controls of whole genome transcript profiles.

Figure S6: PS regulated by TSA treatment

Figure S7: Overlap T4d, T6d and MP treatment

Figure S8: Word clouds of overrepresented GOs affected by 6 h exposure to VPA.

Figure S9: Distinct effects of short and prolonged exposure to HDACi.

Figure S10: Commonly down-regulated oGOs

Figure S11: Primers used for RT-qPCR and Chip

Figure S12: Antibodies used for Western blot and ChIP

Excel Files available online:

Table S1: List of differentially regulated PS for 6h, DoD4 and DoD6

Table S2: List of differentially regulated PS for each treatment condition with TSA and VPA

Table S3: List of oGOs for each treatment condition with TSA

Table S4: List of oGOS for each shown overlap analysis

A No increase in promoter histone acetylation by HDACi used in this study

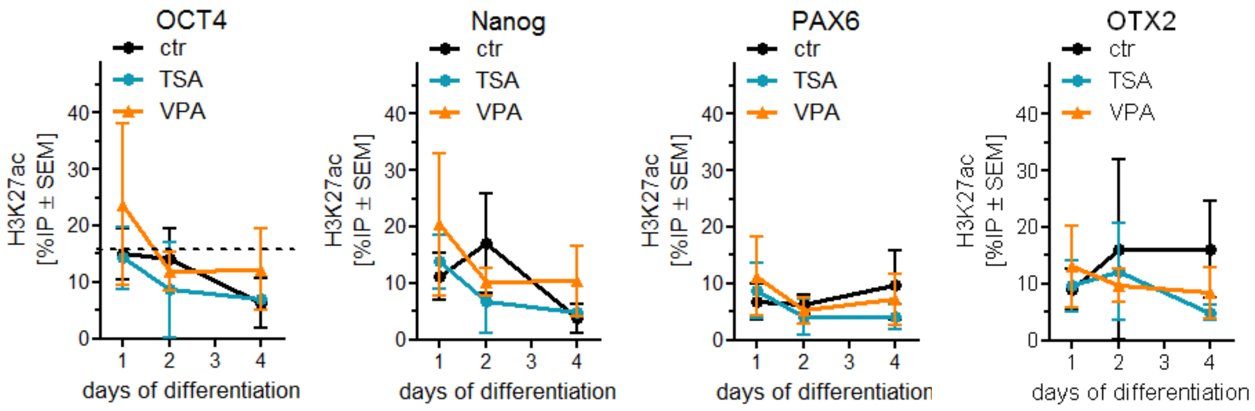

B Increase of promoter acetylation by high concentrations of HDACi

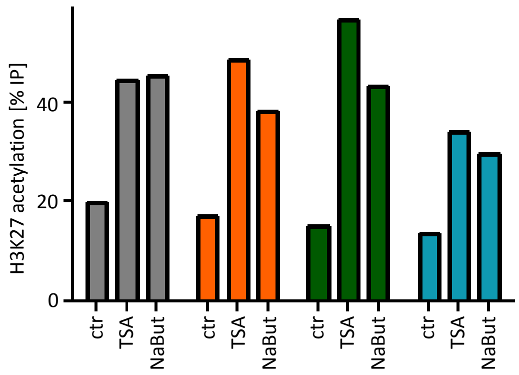

Figure S1: Experimental controls for histone acetylation analysis

Chromatin immunoprecipitation (ChIP) of H3K27 acetylation at marker gene promoters. (A) Histone acetylation at promoters of indicated marker genes for neural differentiation. ChIP was performed with antibodies specific for H3K27ac on samples differentiated in presence or absence of trichostatin A (TSA) and valproic acid (VPA) for indicated time periods. The amount of DNA from the promoter region of indicated genes was quantified by qPCR and compared to control precipitates to obtain enrichment factors (EF) of the chromatin mark which is displayed as % of IP from at least 3 independent experiments. (B) ChIP on samples treated for the first 6 h of differentiation with 50 nM TSA (normally 10 nM TSA were used), or a combination of 10 mM sodiumbutyrate and 10 mM nicotinamide and subsequently lysed. These samples are used as positive control for the antibody against H3K27ac, as the high concentrations of HDAC inhibitors should result in an increase of histone acetylation, which can also be seen compared to the control at every investigated promoter.

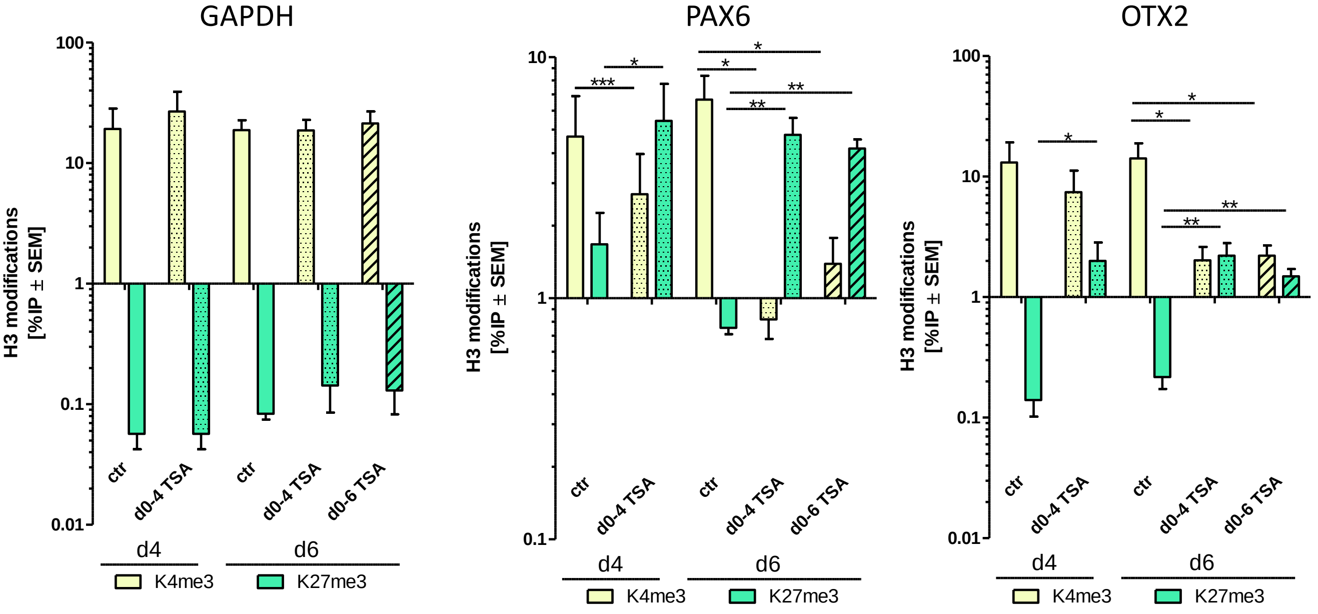

Figure S2: Details on histone methylation changes as basis for Fig. 1E.
Chromatin immunoprecipitation (ChIP) was performed with antibodies specific for H3K4me3 or H3K27me3 on samples differentiated in presence or absence of TSA at indicated time periods. The amount of DNA from the promoter region of indicated genes was quantified by qPCR and compared to control precipitates to obtain enrichment factors (EF) given as % IP for the two histone marks. Data are means ± SEM of 3 experiments. Statistical analysis was done by repeated measured ANOVA followed by Tukey's multiple comparison test *p<0.05 **p<0.01 ***p<0.01 vs. control at respective days.

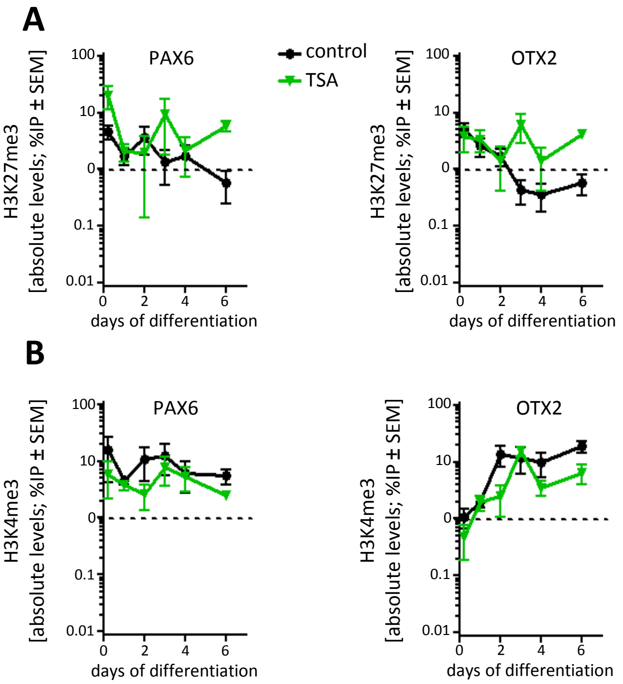

Figure S3: Details on histone methylation changes as basis for Fig. 2A.
Chromatin immunoprecipitation (ChIP) was performed with antibodies specific for H3K4me3 or H3K27me3 on samples differentiated in presence or absence of TSA at indicated time periods. The amount of DNA from the promoter region of indicated genes was quantified by qPCR and compared to control precipitates to obtain enrichment factors (EF) given as % IP for the two histone marks. Data are means ± SEM of 3 experiments. Statistical analysis was done by repeated measured ANOVA followed by Tukey's multiple comparison test *p<0.05 **p<0.01 ***p<0.01 vs. control at respective days.

Figure S3 Balmer et al., 2014

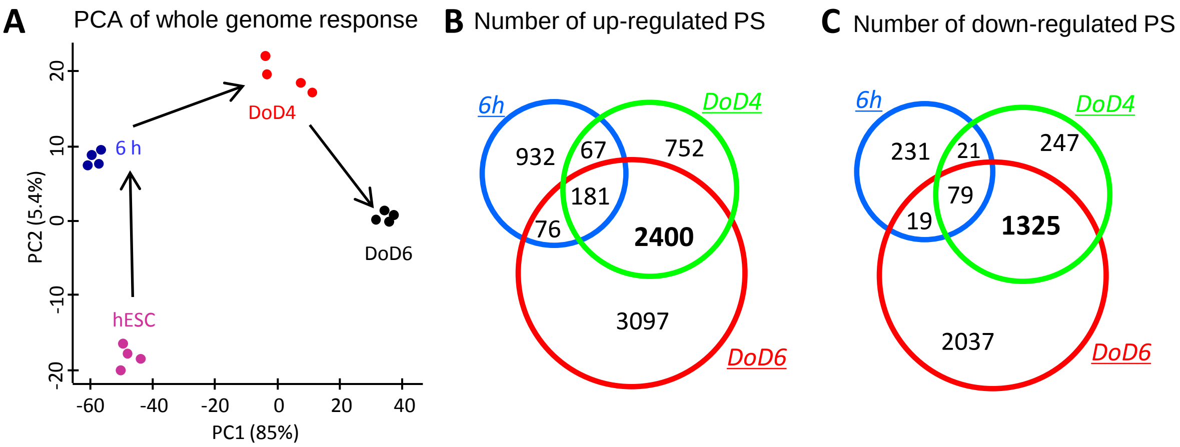

D Gene ontology term (GO) overrepresentation amongst upregulated genes at DoD6, visualized in wordcloud format

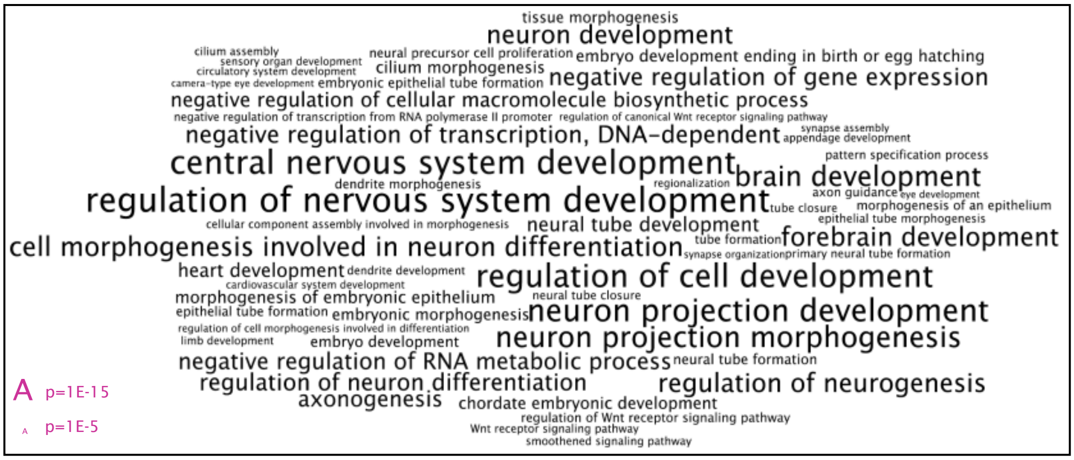

Gene ontology term (GO) overrepresentation amongst downregulated genes at DoD6, visualized in wordcloud format

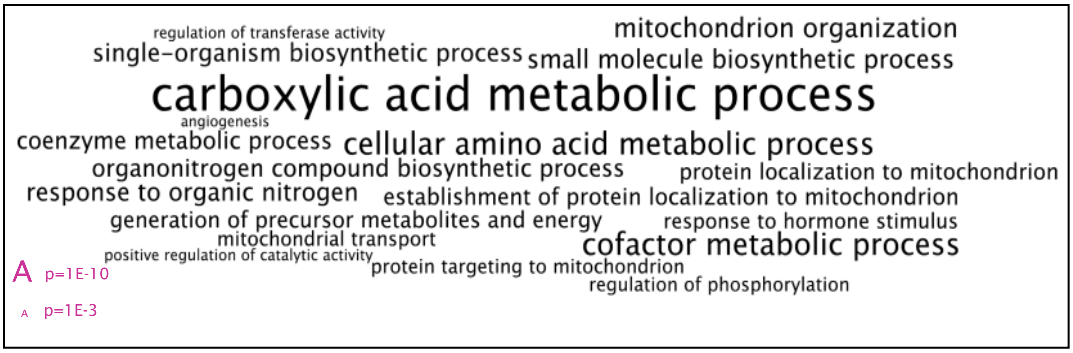

Figure S4: Transcriptome changes during early neuroectodermal differentiation.
(A) Two-dimensional representation of principal component (PC) analysis of all data sets. (B) Comparison of expression changes of up-regulated PS over time. Venn diagrams were constructed for regulated PS (p<0.01, FC>1.5 or FC<0.67). The numbers in the sectors represent absolute numbers. (C) Comparison of expression changes of down-regulated PS over time. Venn diagrams were constructed for regulated PS (p<0.01, FC>1.5 or FC<0.67). The numbers in the sectors represent absolute numbers. (D) Overrepresented gene onthologies (GOs) amongst up- and down-regulated PS at DoD6. The character size in the word clouds is relative to the p-value of the corresponding GO (legends in purple).

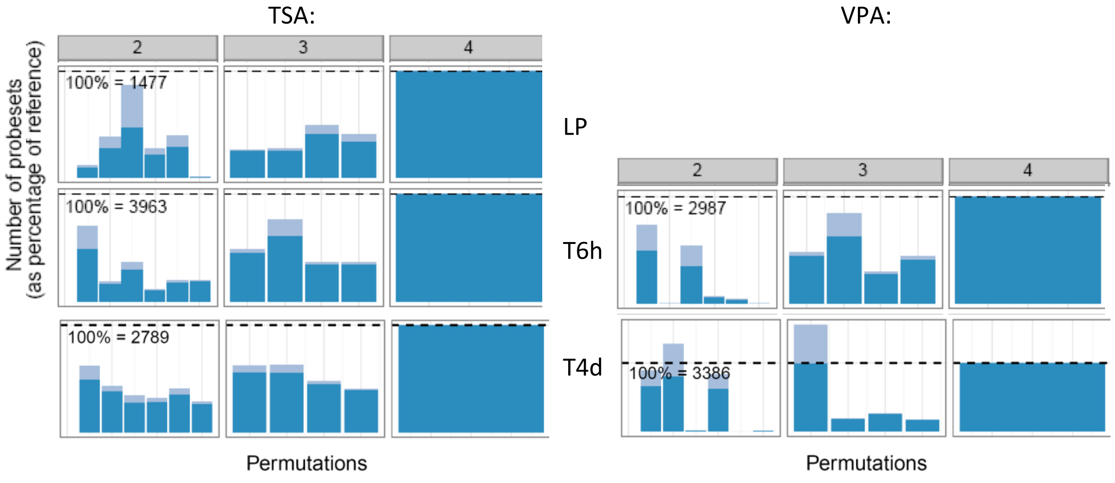

Figure S5: Quality controls of whole genome transcript profiles.
Permutation analysis to control for potential outliers and microarray homogeneity. Permutation analysis of samples incubated for short time and possibly showing heterogeneity. The numbers of significantly altered PS given as 100% are higher than in our normal analysis (see Fig. 2) as no cut-off was set for fold change of regulation. Using all 4 arrays for analysis is set to 100% of significantly regulated PS. Panel 3 shows the number of regulated PS with all 3 possible combinations of leaving 1 array out. Panel 2 shows the number of regulated PS with all 6 possible combinations of leaving 2 arrays out. The analysis shows no outlier in these critical treatment periods.

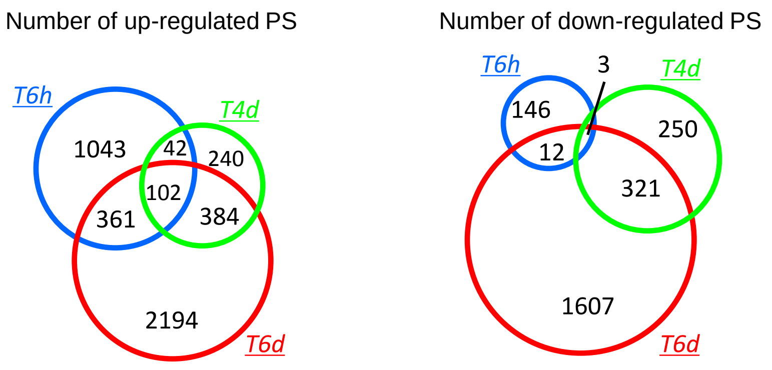

Figure S6: PS regulated by TSA treatment
Venn diagrams displaying actual numbers of PS corresponding to Fig. 3D. hESC were differentiated to NEP and treated and lysed with TSA for the indicated time periods and lysed at DoD6 (T6d), DoD4 (T4d) and after 6 h of induction of differentiation (T6h). The amount of commonly up- and down-regulated PS by TSA treatment performed on regulated PS at indicated time points are displayed as venn diagrams corresponding to Fig. 3D.

Fig. S6 Balmer et al., 2014

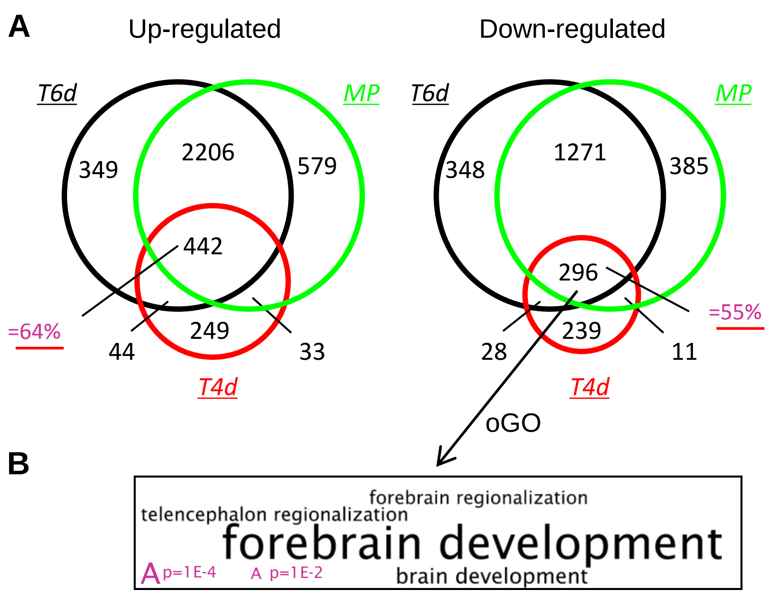

Figure S7: Overlap of 4 day (T4d) treatment and 6 day treatment with (MP) or without (T6d) washout.
(A) Cells were differentiated in presence or absence of TSA for 6 days (T6d) or for 4 days followed by a period of 2 days in absence of the drug (MP) or for 4 days only (T4d). Cells were lysed and genome wide expression profiles were prepared at DoD6 (T6d and MP) or DoD4 (T4d). The numbers of up-regulated (left panel) or down-regulated (right panel) PS is given relative to untreated control. (B) Overrepresented gene ontology terms (GOs) of genes commonly down-regulated by T6d, MP and T4d are displayed as word clouds. The character size corresponds to the p-value. See Tab. S3 for GOs of PS up-regulated by T6d and MP.

VPA (T6h):

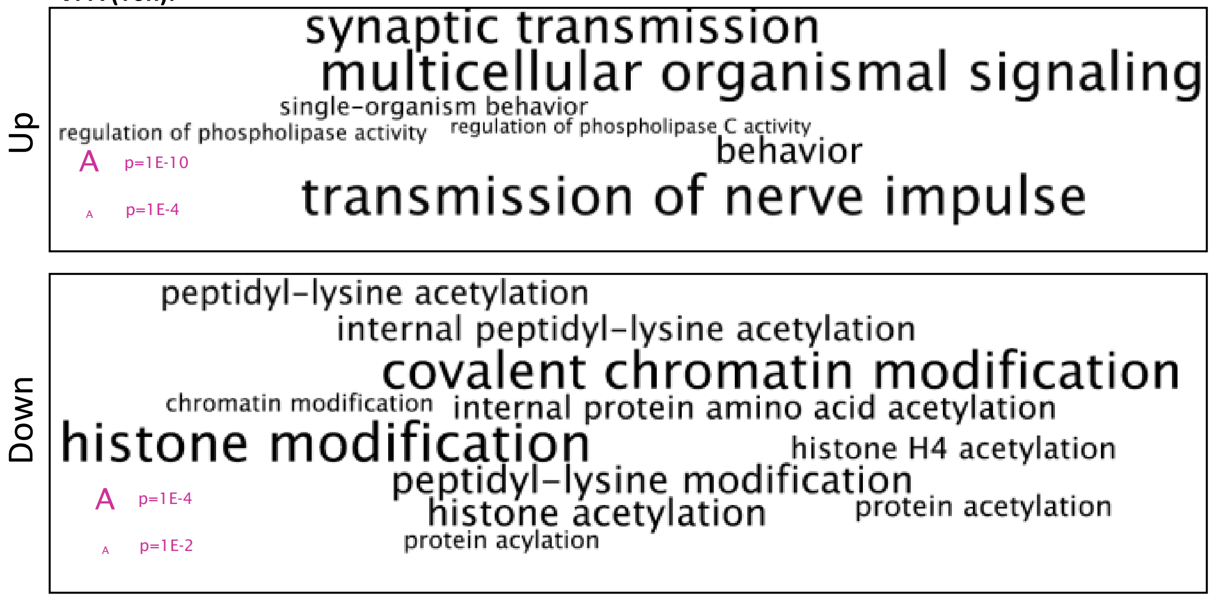

Figure S8: Word clouds of overrepresented GOs affected by 6 h exposure to VPA.
The character size scaling relative to the p-value of the corresponding GO is shown in purple. The regulated PS (up or down) used as input data for the analysis correspond to the effect of TSA by short exposure displayed in Figure 6A

A

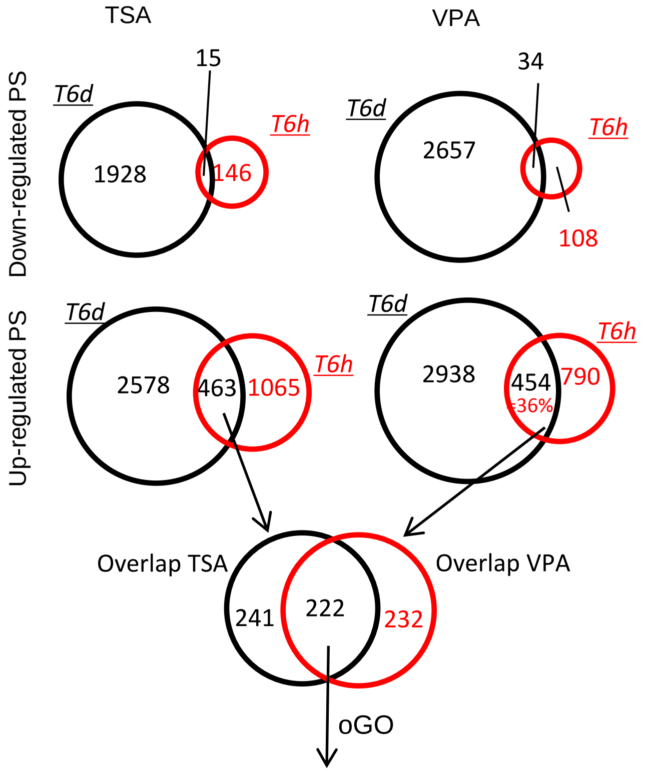

B

P-value	GO term	GO name
1.38e-05	GO:0010517	regulation of phospholipase activity
3.60e-05	GO:0010863	positive regulation of phospholipase C activity
4.06e-05	GO:1900274	regulation of phospholipase C activity
1.23e-04	GO:0010518	positive regulation of phospholipase activity
2.04e-04	GO:0060191	regulation of lipase activity
2.95e-04	GO:0060193	positive regulation of lipase activity
6.48e-03	GO:0014032	neural crest cell development
7.16e-03	GO:0007202	activation of phospholipase C activity
1.50e-02	GO:0014033	neural crest cell differentiation
2.46e-02	GO:0016337	cell-cell adhesion
4.22e-02	GO:0016477	cell migration

Figure S9: Distinct effects of short and prolonged exposure to HDACi.
(A) Comparison of early short (T6h) vs prolonged (T6d) exposure to TSA or VPA. Cells were differentiated for indicated time periods in the presence or absence of TSA (left panel) or VPA (right panel). Differentially expressed PS were determined after long and short exposure to TSA and VPA. The number of regulated PS is given relative to untreated controls. The Venn diagrams display the overlap of up- or down- regulated PS at indicated exposure periods. Then, the PS commonly regulated by T6d and T6h were compared between TSA and VPA. (B) GO terms significantlly overrepresented (oGO) amongst the consensus overlapping PS of VPA and TSA treatments.

Fig. S9 Balmer et al., 2014

oGO among up-regulated PS

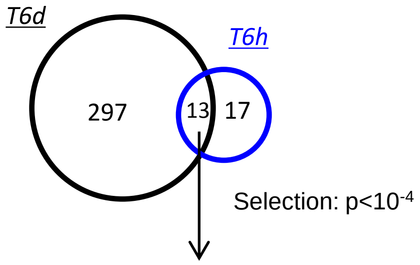

P-value (T6h)	P-value (T6d)	GO term	GO name
1.40e-08	8.93e-03	GO:0007610	behaviour
3.77e-03	1.69e-15	GO:0072359	circulatory system development *
3.77e-03	1.69e-15	GO:0072358	cardiovascular system development *
6.12e-03	2.46e-13	GO:0048514	blood vessel morphogenesis *
1.01e-02	5.50e-15	GO:0001568	blood vessel development *
1.55e-02	5.09e-15	GO:0001944	vasculature development *
2.14e-02	4.30e-09	GO:0001525	angiogenesis *
3.81e-02	6.77e-06	GO:0023056	positive regulation of signaling
7.00e-09	7.52e-03	GO:0035637	multicellular organismal signaling

Figure S10: Commonly oGOs of T6d and T6h among downregulated PS
Comparison of oGOs commonly down-regulated after long (T6d) and short treatment (T6h). (B) Table of oGOs overrepresented both in T6h and T6d.

Fig. S10 Balmer et al., 2014

Supplementary Figure 11
Primers used for RT-qPCR and Chip.

Name	Accession nr.	Forward sequence	Reverse sequence
Primers for gene expression analysis			
**EMX2**	NM_001165924.1	5’-CCAAGGGAACGACACTAGCC-3’	5’-CCATACTTTTACCTGAGTTTCCGTG-3’
**NANOG**	NM_024865.2	5’-GGTGAAGACCTGGTTCCAGAAC-3’	5’-CATCCCTGGTGGTAGGAAGAGTAAAG-3’
**OCT4**	NM_001173531.1	5’-GCAAAGCAGAAACCCTCGTGC-3	5’-ACACTCGGACCACATCCTTCTCG-3’
**OTX2**	NM_014562	5‘-CAG CCC TCA CTC GCC ACA TC-3‘	5‘-GGA GGT GCA AAG TCG GCC CA-3‘
**PAX6**	NM_000280	5‘-CCGCCTATGCCCAGCTTCAC-3‘	5‘-AAGTGGTGCCCGAGGTGCCC-3‘
**RPL13A**	NM_012423.2	5’-GGTATGCTGCCCCACAAAACC-3’	5’-CTGTCACTGCCTGGTACTTCCA-3’
**ZIC3**	NM_003413.3	5’-CTTTGCCCGTTCTGAGAAC-3’	5’-ATGTGCTTCTTACGGTCGCT-3’
**TBP**	NM_001172085.1	5‘-GGGCACCACTCCACTGTATC-3‘	5‘-GCAGCAAACCGCTTGGGATTATATTCG-3‘
Primers for ChIP analysis			
**GAPDH**	NT_009759.16	5‘-TCGACAGTCAGCCGCATCT-3‘	5‘-CTAGCCTCCCGGGTTTCTCT-3‘
**OCT4**	NT_113891.2	5‘-GAGGATGGCAAGCTGAGAAA-3‘	5‘-CTCAATCCCCAGGACAGAAC-3‘
**OTX2**	NT_026437.12	5‘-CAG CAA ATC TCC CTG AGA GCG G-3‘	5‘-GAG GAA GGC GGC TAG AGT TCT AAA C-3‘
**PAX6**	NT_009237.18	5‘-AAGGGAACCGTGGCTCGG-3‘	5‘-ATTAGCGAAGCCTGACCTCTG-3‘
**NANOG**	NT_009714.17	5‘-GTTCTGTTGCTCGGTTTTCT-3‘	5‘-TCCCGTCTACCAGTCTCACC-3‘

Supplementary Figure 12
Antibodies used for Western blot and ChIP.

Antigen	Antibody (supplier)	Catalogue number	Dilution	Species
Antibodies used for western blot				
**H3Ac**	Anti-acetyl-H3 (Millipore)	06-599	1:5000	rabbit
**Pan-H3**	anti-H3 (Abcam)	ab1791	1:5000	rabbit
**α-tubulin**	α-tubulin (Cell Signaling)	2125	1:10000	rabbit
**α-tubulinAc**	Acetyl-α-tubulin(Lys40) (Cell Signaling)	5335	1:10000	rabbit
**PAX6**	anti-Pax6 (Covance)	PRB-278P	1:1000	rabbit
**OTX2**	anti-Otx2 (Millipore)	ab9566	1:1000	rabbit
**Rabbit IgG**	Anti-rabbit-HRP (GE Healthcare)	NA934V	1:10000	donkey
Antibodies used for ChIP				
**H3K4me3**	anti-H3K4me3 (Millipore)	17-614		rabbit
**H3K27me3**	anti-H3K27me3 (Active Motif)	39535		rabbit
**H3K27Ac**	Anti-histone H3 (acetyl K27) (Abcam)	aT2729		rabbit
